# The Role of Non-Coding RNAs in Regulating Cachexia Muscle Atrophy

**DOI:** 10.3390/cells13191620

**Published:** 2024-09-27

**Authors:** Guoming Chen, Jiayi Zou, Qianhua He, Shuyi Xia, Qili Xiao, Ruoxi Du, Shengmei Zhou, Cheng Zhang, Ning Wang, Yibin Feng

**Affiliations:** 1 School of Chinese Medicine, Li Ka Shing Faculty of Medicine, The University of Hong Kong, Hong Kong SAR, China; gmchen@connect.hku.hk (G.C.); zttc@connect.hku.hk (C.Z.); ckwang@hku.hk (N.W.); 2 First Clinical Medical College, Guangzhou University of Chinese Medicine, Guangzhou 510405, China; 2020064220@stu.gzucm.edu.cn (J.Z.); 2022014118@stu.gzucm.edu.cn (Q.H.); 3 Fifth Clinical Medical College, Guangzhou University of Chinese Medicine, Guangzhou 510405, China; 2021081024@stu.gzucm.edu.cn; 4 Second Clinical Medical College, Guangzhou University of Chinese Medicine, Guangzhou 510405, China; 2022024092@stu.gzucm.edu.cn (Q.X.); 2023022054@stu.gzucm.edu.cn (S.Z.); 5 Eighth Clinical Medical College, Guangzhou University of Chinese Medicine, Guangzhou 510405, China; 2022061167@stu.gzucm.edu.cn

**Keywords:** muscle atrophy, cachexia, non-coding RNAs, microRNAs, lncRNAs, circRNAs

## Abstract

Cachexia is a late consequence of various diseases that is characterized by systemic muscle loss, with or without fat loss, leading to significant mortality. Multiple signaling pathways and molecules that increase catabolism, decrease anabolism, and interfere with muscle regeneration are activated. Non-coding RNAs (ncRNAs), such as microRNAs (miRNAs), long non-coding RNAs (lncRNAs), and circular RNAs (circRNAs), play vital roles in cachexia muscle atrophy. This review mainly provides the mechanisms of specific ncRNAs to regulate muscle loss during cachexia and discusses the role of ncRNAs in cachectic biomarkers and novel therapeutic strategies that could offer new insights for clinical practice.

## 1. Introduction

Cachexia is a complex and multifactorial syndrome characterized by loss of skeletal muscle and weight with or without loss of fat mass, associated with various chronic diseases, including cancer, heart failure, kidney disease, chronic obstructive pulmonary disease (COPD), and AIDS [1]. It is reported that approximately 80 percent of cancer patients suffer from cachexia, which contributes to 40 percent of deaths linked to cancer [2]. Muscle atrophy is a prominent feature of cachectic syndrome due to the reduction of muscle fibers and the decrease in muscle mass and strength, relating to poor survival and significantly diminished quality of life for patients [3]. The mechanism of cachexia muscle atrophy is regarded as disordered and imbalanced protein and energy metabolism, which also impacts the functions of muscle repair and regeneration [4]. Meanwhile, inflammatory factors in cachexia can also promote the breakdown of muscle proteins [5].

The vast majority of DNA sequences in the human genome do not code for proteins, with about 80% of human genomic DNA transcribed into RNA, of which only 2% is translated into proteins, and most of the rest is classified as non-coding RNAs (ncRNAs) [6]. Long non-coding RNAs (lncRNAs), circular RNAs (circRNAs), and microRNAs (miRNAs) have all been demonstrated to have important regulatory functions in the physiological and pathological processes of cancer cachexia in recent years [7,8,9]. Additionally, emerging studies have shown that ncRNAs are an indispensable regulator of muscle atrophy [10,11,12].

However, a more extensive understanding of ncRNAs in cachexia muscle atrophy is necessary. Therefore, this review will overview the mechanisms of ncRNAs through multiple signal pathways in muscle atrophy caused by cachexia and describe the prognosis indicators and gene therapy, with the hope of expanding our knowledge of cachexia muscular atrophy and offering potential approaches for clinical practice.

## 2. Mechanisms of Non-Coding RNA for Muscle Protein Synthesis

### 2.1. IGF-1-PI3K/AKT-mTOR Pathway

The main etiology of cachectic muscle atrophy is a breakdown of protein metabolism, characterized by insufficient protein synthesis and excessive proteolysis in muscle [13]. The insulin-like growth factor 1 (IGF-1)-PI3K/AKT-mTOR pathway primarily controls protein synthesis. Following binding with IGF-1, the insulin receptor substrate1 (IRS1), an intracellular adaptor protein, recruits PI3K and Akt protein, vital for skeletal muscle regeneration [14]. As a downstream target of Akt, mTOR is tightly associated with multiple biological processes, such as protein synthesis, nucleic acid synthesis, glucose metabolism, and ATP generation. There are two complex forms of mTOR: mTORC1 and mTORC2. When amino acid levels are high, mTORC1 is activated to induce the phosphorylation of eukaryotic translation initiation factor 4e-binding protein (4E-BP) and S6 kinase (S6K), thus promoting protein synthesis and cell growth [14,15]. Therefore, the IGF-1/Akt/mTOR pathway is critical in regulating protein metabolism in target therapy for cachexia.

**Figure 1:**
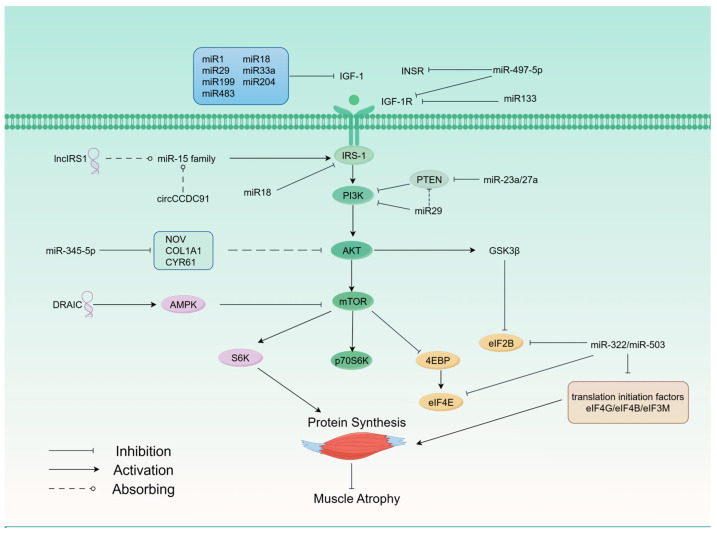
Overexpressed miR1, miR18, miR29, miR33a, miR199, miR204, miR483 can inhibit IGF-1—PI3K—AKT—mTOR pathway by targeting IGF-1. lncIRS1 rescues muscle atrophy by upregulating the expression of its target gene, IRS1, with the sponge of the miR15 family. miR18 can also inhibit IRS-1 to regulate the IGF-1 pathway. DRAIC can regulate the translation of proteins during tumorigenesis by influencing the AMPK-mTOR-S6K signaling pathway. Overexpression of miR-322/miR-503 targets translation initiation factors specifically to decrease protein translation, leading to muscle atrophy.

Non-coding RNAs have been implicated in regulating the IGF-1-PI3K/AKT-mTOR pathway in muscle, with some exerting upregulating effects while others exhibit down-regulating effects. We have summarized the regulation of cachexia by relevant ncRNAs through the IGF-1-PI3K/AKT-mTOR pathway (Figure 1, Table 1).

#### 2.1.1. ncRNAs That Inhibit IGF-1-PI3K/AKT-mTOR Pathway

Recent research has shown that miR-204 and miR-33a can suppress the expression of IGF-1 and hence impede the PI3K/Akt-mTOR signaling pathway. It is found that miR-204 can inhibit cell growth and differentiation by targeting IGF-1 3′ UTR regions in C2C12 myoblasts while downregulated following damage to the skeletal muscle in vivo [16]. MiR-204-5p was proven to regulate the leptin signaling pathway and accelerate the degradation of white adipose tissue to deteriorate cachexia in cancer patients [17]. However, there is currently no relevant study on the regulation of miR-204 in skeletal muscle in the cachexia model. Since the molecular target of miR-33a/b is the IGF-1 receptor, the high circulation of miR-33a/b can also block the IGF-1-PI3K signaling pathway [18]. In the duck myoblast model in vitro, overexpression of miR-33a inhibited the expression of Akt/p-Akt, mTOR/p-mTOR, and p-S6K after transfection.

According to the research by Martin Connolly et al., miR-424-5p could oppose protein synthesis and induce muscle loss in COPD or ICU-acquired weakness patients, due to preventing rRNA synthesis through binding the Pol I pre-initiation complex [19]. It is noticed that in muscle biopsies from cachectic non-small cell lung cancer (NSCLC) patients, the level of miR-424-5p is increased compared with control groups [20]. Interestingly, recent studies have reported that miR-424-5p can target PI3K/Akt signaling to accelerate the development of some types of cancer [21,22,23], but whether the enhancement of cancer cachexia induced by miR-424-5p has a definite connection with the PI3K/Akt signaling pathway is still unknown. Besides, a miRNAs sequencing test has shown that miR-424-5p level significantly increases in Duchenne muscular dystrophy (DMD) patients and is related to the severity of muscle loss [24].

The Cyr61/CTGF/NOV (CCN) proteins are extracellular matrix proteins, which affect cell proliferation, migration, and tissue repair [25]. It is found that NOV and CYR61 participate in the IGF-1 signaling through the AKT and mTOR pathway [26]. In the analysis of miRs in the skeletal muscle of cancer patients with cachexia, miR-345-5p is up-regulated. In contrast, the muscle transcriptome dataset reveals the upregulation of CYR61 and the downregulation of its targets NOV and COL1A1 [27].

According to earlier research, miR-483 targets IGF-1 signaling and suppresses the expression of several crucial proteins in the PI3K/AKT signaling pathway (IRS1, PI3K, PDK1, and AKT) [28], which control the proliferation and development of bovine myoblast cells. However, additional research is necessary to determine whether miR-483 has comparable effects on human muscle, given that the investigations have merely relied on samples from bovine skeletal muscles.

According to the experimental findings, transfection of miR-29b mimics into C2C12 myotubes decreased IGF-1 signaling and PI3K (p85a) protein levels as well as phosphorylation of downstream effectors of IGF-1, including mTOR, AKT, and P70S6K. Therefore, miR-29b targets PI3K and IGF-1 to promote various muscular atrophy in vivo and in vitro, such as in mouse models with cancer, aging, TNF-α, or denervation [29]. Notably, the inhibition of miR-29b can bring hope to therapy for cachexia muscle atrophy.

Diabetic cardiomyopathy (DCM) is a common complication of diabetes, independent of hypertension and coronary artery diseases, and is mainly characterized by cardiomyocyte hypertrophy, which can eventually lead to heart failure [30]. In the mouse model with diabetes, the overexpression of miR-203 is found to target the PIK3CA gene to inhibit the PI3K/Akt signaling pathway, attenuating cardiac hypertrophy. It is suggested that miR-203 might serve as a cardioprotective regulator in DCM to provide potential targeted therapeutic options for it [31].

#### 2.1.2. ncRNAs That Activate IGF-1-PI3K/AKT-mTOR Pathway

It has been previously suggested that non-coding RNAs can also positively affect the IGF-1 signaling pathway, therefore antagonizing muscle wasting. Phosphatase and tensin homolog (PTEN), a molecular regulator that inhibits the phosphorylation of PIP2 to PIP3, can prevent the activation of the AKT/mTOR pathway [32]. In skeletal muscle, muscle atrophy F-box (MAFbx) and muscle RING finger 1 (MuRF1) stand out as the prototypical E3 ubiquitin ligase, which promotes atrophy-associated protein degradation and enables these proteins to be degraded through the 26S proteasome [33].

In neonatal rat cardiomyocytes, miR-486 inhibits PTEN expression that activates AKT/mTOR signaling, promoting protein synthesis. Consequently, the downregulation of miR-486 elevates PTEN and FoxO1 expression, increasing Atrogin1 and MuRF1 levels in vivo [34]. Injection of AAV-miR-23a/27a/24-2 in mice with muscle atrophy induced by chronic kidney disease (CKD) could increase phosphorylation of Akt and FoxO and reduce muscle loss, meanwhile increasing grip strength and, interestingly, inhibiting PTEN expression [35].

In gastrocnemius atrophy of rats with diabetes, exogenous miR-23a/27a can inhibit the myostatin cascade and upregulate the insulin signaling pathway, leading to reduced muscle loss and improved muscle function [36]. Earlier research found that calcineurin (Cn) can improve miR-23a expression by activating T cell nuclear factor 3 (NFATc3) in a rat model of cardiac hypertrophy [37]. Still, overexpression of Cn cannot elevate the miR-23a levels in skeletal muscle [33]. However, it has been proven that Cn expression could control the decrease inof miR-23a in the muscle atrophy model induced by Dex [36]. Meanwhile, IGF-I could increase the Cn levels and elevate NFATc1 expression, leading to muscle growth [38]. The complicated relationship between miR-23a, calcineurin, and NFAT in different types of muscle tissues and muscle atrophy or hypertrophy models, as well as the effect of the IGF-1 pathway in them, need further investigation, with the hope of providing more available methods for muscular atrophy therapy.

The co-transfection experiments revealed that circRILPL1 effectively counteracted the inhibitory influence of miR-145 on the target gene IGF-1R. Remarkably, circRILPL1 was found to act as a miR-145 sponge, promoting the IGF-1R/AKT/PI3K signaling pathway’s activation [39]. Consequently, this activation enhanced muscle proliferation, elevated cell differentiation, and reduced muscle loss.

Recently, it was discovered that circCCDC91 directly binds to the miR-15 family in chicken’s skeletal muscle, promoting myoblast development and proliferation while reducing muscle atrophy. By adsorbing miR-15c-5p, miR-15b-5p, and miR-15a, circCCDC91 regulates the expression of IRS1 and triggers the IGF-1-PI3K/AKT pathway [40]. Interestingly, a newly identified lncRNA called lncIRS1 is also a competing endogenous RNA (ceRNA) in the miR-15 family, acting as a molecular sponge for miR-15c-5p/miR-15b-5p/miR-15a to raise the expression of the IRS1 gene, resulting in the enhancement of the phosphorylation level of AKT in myoblasts and promoting muscle abundance in broilers [41].

In summary, non-coding RNAs can regulate multiple steps in the IGF-1-PI3K/AKT-mTOR pathway, such as CCN proteins, PTEN, FoxO, and MuRF1 expression, to control protein synthesis or degradation rates and thus muscle mass. Targeting specific ncRNAs could be a potential therapy to help counter muscle loss in cachexia.

**Table 1:** Roles of non-coding RNAs in muscle protein synthesis in muscle atrophy by regulating IGF-1-PI3K/AKT-mTOR pathway.

ncRNAs	Type of Study	Time	Expression	Biological Significance	References
miR-204	In vitro: C2C12 myoblasts In vivo: the CTX-injured mouse TA muscle	48 h; 21 d	Inhibited IGF-1, Pax7, and Mef2c expression; downregulated miR-204 after muscle injury	Downregulated proliferation, migration, and differentiation in C2C12 myoblasts	[16]
miR-33a	In vitro: duck myoblasts	36 h	Inhibited IGF-1 expression and blocked PI3K/Akt/mTOR signaling	Suppressed Akt, p-Akt, mTOR, p-mTOR, S6K, and p-S6K protein expression after transfection	[18]
miR-1	In vitro: C2C12 myoblasts	/	Inhibited IGF-1 expression	Downregulated miR-1 results in increased IGF-1 and Akt protein level	[42]
miR-133	In vitro: C2C12 myoblasts	8 d	Inhibited IGF-1R expression	Suppressed IGF-1/PI3K/Akt signaling in a negative feedback circuit	[43]
miR-206	In vitro: mice muscle with denervated atrophy	0, 7, 14, 28 d	Inhibited IGF-1 mRNA expression	Improved muscle atrophy of denervated slow and fast muscles	[44]
miR-199	In vitro: Piaractus mesopotamicus fast and slow muscle fibers	/	Inhibited IGF-1 and mTOR expression	Downregulated IGF-1 and mTOR mRNA expression	[45]
miR-345-5p	In vitro: skeletal muscle from CC and NCC patients	/	Downregulated NOV and upregulated CYR61	Inhibited IGF-1 and AKT/mTOR signaling and suppressed muscle protein synthesis	[27]
miR-486	In vivo: miR-486 transgenic TA muscle after CTX injury and wild-type mice	5 d	Decreased levels of PTEN mRNA	Upregulated the IGF-1/Akt pathway, reduced muscle loss, and improved grip strength	[46]
	In vitro: C2C12 cells In vivo: myostatin knockout mice	5 d	Increased miR-486 expression in myostatin knockout mice	Myostatin attenuated the IGF-1/Akt pathway by repressing miR-486 expression and downregulated skeletal muscle size	[47]
miR-497-5p	In vitro: C2C12 myotubes	15 h	Inhibited IGF-1r and Insr	Decreased the myotubes size	[48]
miR-483	In vitro: bovine myoblast cells	24 h	Inhibited the IGF-1/PI3K/AKT signaling pathway	Prevented bovine myoblast cell proliferation and differentiation	[28]
miR-29b	In vivo: mice with six types of muscle atrophy In vitro: C2C12 myoblasts	2 wk; 6 d	Inhibited the IGF-1/PI3K/AKT signaling pathway	Promoted various types of muscular atrophy	[29]
miR-23a/ miR-27a	In vivo: CKD mice In vitro: C2C12 myotubes	3 d	Increased the phosphorylation of Akt and decreased PTEN and FoxO1	Improved CKD-induced muscle atrophy	[35]
miR-203	In vivo: DM mice	3 d	Inhibited PI3K/Akt signaling pathway by targeting PIK3CA	Reduced myocardial hypertrophy induced by myocardial fibrosis	[31]
lncIRS1	In vitro: chicken primary myoblasts In vivo: pWPXL/pLKO1- lncIRS1 chicks	/	Increased IRS1 abundance and promoted the phosphorylation of AKT, as a sponge for the miR-15 family	Promoted skeletal muscle myogenesis and improved atrophy	[41]
CircRILPL1	In vitro: bovine primary myoblasts	4 d	Inhibited miR-145 and upregulated the IGF-1-PI3K/AKT pathway	Improved muscle atrophy	[39]
Circ CCDC91	In vitro: Dex-induced chicken myoblasts	3 d	Activated the IGF-1-PI3K/AKT pathway	Upregulated the protein levels of AKT/p-AKT and Atrogin-1, improved skeletal muscle atrophy	[40]

### 2.2. AMPK–mTOR Pathway

As a cellular energy sensor, AMP-activated protein kinases (AMPKs) promote the ATP catabolic pathway while inhibiting energy expenditure to maintain energy balance in cells when faced with energy challenges [49]. It is found that AMPK attenuates mTORC1, which contributes to inhibiting protein synthesis and cell growth [50]. AMPK phosphorylates tuberous sclerosis complex 2 (TSC2) to inhibit Ras homolog enriched in brain (RHEB), which is a positive regulator of mTORC1. Meanwhile, AMPK can phosphorylate the regulatory associated protein of mTOR (RAPTOR) to inactivate mTORC1 [49].

As a long non-coding RNA, low expression of DRAIC was a marker for poor prognosis for these patients in seven different malignancies and gliomas [51]. Shekhar Saha and colleagues reported that DRAIC exerts its tumor suppressive function by modulating Glucose transporter 1 (GLUT1) expression to deliver signals from IKK/NF-κB to the AMPK/mTOR pathway. The inhibition of mTOR by DRAIC results in the suppression of protein translation, cell invasion, and autophagy activation [51]. Besides, DRAIC stimulates AMPK and can prevent the phosphorylation of key substrates by mTORC1 [51], thereby providing a therapeutic approach for the AMPK–mTOR pathway regulation of tumor-induced cachexia muscle atrophy.

### 2.3. Eukaryotic Translation Initiation Factors

Eukaryotic translation initiation is a complicated and essentially controlled process that involves many eukaryotic initiation factors (eIFs) that assemble at the 5′ cap structure of the mRNA to recruit the 40S ribosomal subunit [52]. This rate-limiting step determines the protein synthesis rate. eIF4F complex assembly is a key checkpoint targeted by ncRNAs. It consists of the scaffolding protein eIF4G, the RNA helicase eIF4A, and the cap-binding protein eIF4E. In particular, eIF4E availability is regulated by 4E-BPs that sequester eIF4E [53].

It has been reported that H19X-encoded miRNAs, such as miR-542 and miR-424-5p, exert important effects on regulating the progress of some cachexia diseases [19,54]. Rui Liang et al. showed that in the mouse genetic models, overexpression of miR-322/miR-503 in the family of H19X miRNAs was found to be a key factor in the stunting of skeletal muscle growth [54]. In the study, the muscle weight in miR-322/-503 transgenic mice was only 54.5% of their littermates of wild type. Oppositely, transcriptional disruption at the H19X gene promoted muscle growth by 14.4–14.9% and decreased the loss of skeletal muscle mass. They ultimately found that miR-503/miR-322(424) encoded by H19X directly manages the amount of translation-initiating factors, eIF4E, eIF4G1, eIF4B, eIF2B5, and eIF3M, to increase protein production and control skeletal muscle atrophy [54].

Patricia S. Pardo et al.’s luciferase assay experiments validated that the mRNA of eIF5A1 is a target of miR-434-3p based on mutations in binding sites [55]. When miR-434-3p is overexpressed in myocytes, it suppresses eIF5A1 in response to diverse apoptotic stimuli, which in turn reverses the situation of reduced mitochondrial transmembrane potential and activation of caspases-3, -8, and -9. Researchers hypothesize that miR-434-3p may have therapeutic value in treating muscle wasting induced by various pathophysiological diseases, due to high levels of eIF5A1 in aging mice’s skeletal muscle [55]. However, there is currently no conclusive experimental proof supporting the use of miR-434-3p for treating cachexia muscular atrophy, and related studies are expected to be conducted in the future.

In vivo, it is found that miR-21 and miR-206 play an important role in the atrophy progress in several muscle-wasting models, including cachexia induced by colon carcinoma [56]. MiR-21 attenuates the transcription factor YY1 and eIF4E3 levels, while miR-206 downregulates the activity of eIF4E3 to strengthen muscle atrophy. YY1 has been proven to upregulate in multiple types of cancer, modulating mitochondrial energy metabolism and enhancing cachectic muscular atrophy [57].

Besides, a previous study has shown that miR-199a-3p regulates the eIF4EBP1 gene to lower mTOR pathway activity in human skeletal muscle with cancer cachexia, interfering with protein synthesis and promoting atrophy [27]. Furthermore, it is revealed that miR-199a is a potential miRNA that prevents cancer cachexia by suppressing the expression of JunB and Caveolin 1 protein, which is involved in the proliferation and metastasis of tumor cells [9].

### 2.4. SOX6

AngII, H_2_O_2_, and TNF-α-induced muscle atrophy are among the several types of muscle atrophy in vivo and in vitro. A recent study has revealed that they have the same common regulator: an lncRNA called muscle-atrophy-associated transcript, or MAAT [58,59]. Overexpression of lncMAAT can prevent multiple types of muscle atrophy, while downregulation of lncMAAT is sufficient to cause atrophy. Mechanistically, lncMAAT uses a trans-regulatory module to negatively regulate the intranuclear transcription of miR-29b via the sex-determining region Y-box 6 (SOX6) and a cis-regulatory module to augment the expression of the nearby gene Mbnl1 [58]. We mentioned that miR-29b is a key microRNA to modulate PI3K and IGF-1 signaling, deteriorating atrophy in various models in Section 2.1.1 [29].

## 3. Mechanisms of Non-Coding RNA for Muscle Protein Degradation

### 3.1. PI3K-AKT-FOXO Pathway

The essential protein degradation pathways are the ubiquitin-proteasome system (UPS), autophagy-lysosome system (ALS), and caspase system. As the core member of the UPS family, both MuRF-1 and MAFbx/Atrogin-1 facilitate proteolysis in muscle and exhibit an elevation in diverse conditions that contribute to muscle atrophy, including chronic diseases like cancer and COPD, which are associated with cachexia [15]. Besides, FOXO transcription factors can activate UPS and ALS, stimulating the expression of MuRF-1 and Atrogin-1, which is vital to evoke muscle protein catabolism during muscle atrophy [60].

Akt is the downstream protein for PI3K signaling, which can control protein synthesis and degradation. AKT phosphorylates FoxO to inhibit its transcription activity and thus reduces the degradation of muscle proteins [61]. In the second chapter, we summarize the ncRNAs associated with protein synthesis, then we review the ncRNAs involved in protein catabolism during muscle atrophy.

Chuncheng Liu et al. [62] have reported that miR-18a directly inhibits IGF-1 mRNA expression in vivo and vitro, reduces the phosphorylation of Akt and FoxO3, and thus activates MuRF1 and Atrogin-1 levels, contributing to muscle atrophy as a regulator of protein catabolism. Besides, it is found that the elevation of miR-21 is associated with FOXO3 nuclear localization. When miR-21 expression is upregulated in myoblasts, FOXO3 translocates into the nucleus, triggering the cellular stress response and apoptosis, which prevents muscle regeneration [63].

Bin Wang et al. have demonstrated that multiple catabolic responses can be reversed by miR-23a/27 [35]. Through elevating PTEN 3′ UTR and FOXO1 expression, miR-23a and miR-27 decrease the activation of AKT, FBXO32/atrogin-1, and MuRF1. In addition, proteolysis by caspase-3 is an additional mechanism contributing to muscle atrophy in CKD, and miR-23a/27a can downregulate AKT to reduce caspase-3 activity, resulting in muscle atrophy alleviation [35].

We have mentioned that miR-29b targets PI3K(p85a) and 3′ UTR of IGF-1, thereby impairing proliferation of muscle progenitor cells [29]. Moreover, a study showed that through the PI3K-AKT pathway, miR-29b might restrain FOXO transcription factors and induce expressions of ubiquitin ligases in NSCLC patients, which is significant for patients’ survival [64].

Besides, in LPS-induced muscle atrophy mice, miR-1290 can downregulate the expression of MuRF1 and atrogin-1 by targeting Akt/p70/FoxO3 signaling, improving the atrophy. Notably, the miR-1290 expression in muscular atrophy patients was greatly lowered compared to normal level [65].

### 3.2. NF-κB Pathway

Muscle atrophy in cancer is also influenced by the transcription factor NF-κB, an important mediator of inflammatory reactions and apoptosis [66]. NF-κB has been linked to the activation of skeletal muscle breakdown and particular regulatory transcription that inhibits IGF-1 muscle-building signaling that responds to the TNF-α pathway [67]. MyoD, a muscle-regulatory factor, is likewise restricted at the transcriptional level by NF-κB in cancer cachexia after TNF-α activation [68]. Apart from the TNF-α signaling, NF-κB is known for stimulating higher MuRF-1 production by triggering the proteolysis of skeletal muscle proteins. Furthermore, upregulated NF-κB-inducible kinase (NIK) advances Atrogin-1, a muscle-wasting element, augmenting the muscle proteolysis [69].

Previous research indicated that in mice with CKD or cancer and fasting models, lncRNA Atrolnc-1 enhances muscle atrophy. Overexpression of Atrolnc-1 stimulates the breakdown of proteins in cultured C2C12 myotubes, whereas knockdown of Atrolnc-1 dramatically reduces the rate of protein degradation accelerated by serum deprivation. The inhibition of ABIN-1, a protein that inhibits NF-κB signaling, is hindered by Atrolnc-1, leading to increased NF-κB reactions and MuRF-1 expression [70].

Besides, it was shown that in the cells’ lines of cisplatin-induced bladder cancer, the activity of EGFR (epidermal growth factor receptor) and NF-κB signaling was enhanced, and the expression of ProT was increased. Moreover, ProT and lncRNA HOTAIR transcription exhibited favorable relationships. Downregulation of EGFR or ProT, or inhibition of the NF-κB pathway, led to lower expression of lncRNA HOTAIR, impeding muscular wasting in cachexia with the inflammatory environment [71].

Furthermore, Endothelin-1 stimulation was the most effective in suppressing MiR-let-7g-5p, which combines with the 3′ UTR binding site of NF-kB and controls both IL-6 and TNF. Inflammation in the microenvironment and injury to fat cells and skeletal muscles could be caused by ET-1 [72].

NFKB1 (referred to as p50), a component of the transcription factor NF-κB, named NFKB1, may particularly bind to the promoter region of miR-532-3p and suppress its production, increasing BAK1 (BCL2 antagonist/killer 1). Muscle atrophy was ultimately induced by the apoptotic effects of the accumulation of BAK1 [73]. A study involving 103 COPD patients found a positive connection between muscle nuclear factor κB p50 and plasma miR-499 in early-stage COPD patients but not with p65. Interestingly, the levels of miR-206 and miR-133 in severe patients are positively correlated with plasma inflammatory cytokines TNF-α, IL-2, and IL-5, indicating that more advanced disease is linked to increased levels of specific miRs and circulating cytokines [74].

### 3.3. IL-6-JAK-STAT3 Pathway

It is reported that via directing the inflammatory response, the cachexia is significantly influenced by IL-6/JAK/STAT3 signaling [75]. STAT3 phosphorylation, triggered by IL-6 binding to the receptor, results in muscle atrophy and proteolysis of skeletal muscle. Specifically, the activity of STAT3 increases the expression and activity of CCAAT/enhancer-binding protein δ (C/EBPδ), which stimulates MURF1, MAFbx, and myostatin levels. The prototype of cachexia triggered by IL-6 showed that skeletal muscle atrophy was prevented by blocking JAK/STAT3 signaling [75].

According to a study, the RNA-binding protein HuR stimulates the activity of STAT3 in the muscle atrophy model, whereas upregulated miR-330 dramatically lowers the STAT3 signaling. Interestingly, it is found that HuR interacts directly with STAT3 mRNA-3′UTR, which is close to the miR-330 seed element, resulting in obstructing the translation regulated by miR-330, implying that their rival relation could offer novel therapy alternatives for STAT3-induced muscle atrophy [76].

MiR-21 is upregulated by TNF-α and IL-6 and has a role in regulating myoblast viability and differentiation, which in turn controls the SCs’ ability to generate novel myotubes. To confirm that the downregulation of miR-21 is a potential strategy for muscle atrophy, more in vivo studies on miR-21 maintaining muscle mass are needed [63]. Furthermore, IL-6 decreases miR-497-5p level as a feedback loop, causing the stimulation of IGF-1r and Insr that link to hypertrophy to offset atrophy [48].

A recent study has demonstrated that an increased miR-5682 level was related to serious malnutrition in laryngeal cancer (LC) patients treated with radiotherapy [77]. MiR-5682 was reported to involve the regulation of the IL-17 pathway in the inflammatory microenvironment [78], and IL-17 enables triggering JAK/STAT3 signaling to aggravate muscle loss [79]. However, the mechanism of how miR-5682 enhances LC-induced muscle atrophy needs to be explored further.

Besides, a study found that the level of miR-27b-3p was reduced significantly while IL-15 expression was elevated in muscle tissue from cancer cachexia patients, and miR-27b-3p was proven to target the IL-15 gene [80]. Previous studies have reported that IL-15 can downregulate protein degradation in skeletal muscle from hepatitis-induced cachexia rats [81], regarded as an approach for the body to antagonize muscle atrophy during cachexia [82].

### 3.4. TNF-α Pathway

As an inflammatory factor, it is stated that TNF-α is necessary for cachexia-induced muscular atrophy. It has been documented that TNF-α directly impacts the breakdown of skeletal muscle by causing the UPS to express ubiquitin genes [83]. Furthermore, TNF-α was substantially engaged in the induction of NF-κB and routinely triggers the activation of the p38 MAPK pathway [67].

A recent study has shown that miR-155 expression is increased in cachexia patients suffering from NSCLC or pancreatic cancer. As a factor that promotes cachexia, miR-155 targets upstream genes of the TNF-α pathway to accelerate the procedure of muscular atrophy [84].

In an NF-κB-dependent way, TNF-α decreased the differentiation of C2C12 myoblasts and impeded myogenic miRNA production. Upregulation of miR-1, miR-133a/b, or miR-206 alleviated this TNF-α-induced halt in differentiation [85]. In another study including 218 participants, of whom half were sarcopenia patients, the decrease in miR-133b and miR-206 expression was shown to have a strong correlation with the development of muscular weakness influenced by the regulation of IL-6 and TNF-α [86].

Ccdc41os1, Gm4117, and 5830418P13Rik were identified as essential lncRNAs for TNF-α-induced myotube atrophy based on a bioinformatics study. Furthermore, Gm4117 contributes to mediating the TNF and FoxO expression in muscular atrophy, as the sponge of the miRNA-467/669 family [87].

A genomic profiling investigation revealed a reduction in miR-23, miR-27, miR-1, miR-133a/b, miR-206, miR-93, and miR-107 due to TWEAK (TNF-like weak inducer of apoptosis) mediation, resulting in muscular dystrophy through several signaling pathways like TGF-β (transforming growth factor-β) and NF-κB [48].

### 3.5. Myostatin

Myostatin, a TGF-β family member expressed in skeletal muscle, has a negative regulatory effect on the growth and development of muscle cells [15]. Besides, myostatin combines with the activin receptor type IIB (ACTRIIB), playing a role as a transmembrane kinase receptor [88], which shows high expression in mammalian skeletal muscle [89]. Moreover, myostatin promotes Smad2/3 signaling to regulate FoxO1 and atrogin-1, resulting in the occurrence of muscle atrophy [90]. Furthermore, myostatin can break the IGF-1-Akt-PI3K pathway, activating FoxO, MAFbx, and MuRF1 proteins [91]. Thus, the myostatin pathway contributes greatly to cachexia-associated muscle atrophy via interaction with ACTRIIB, which involves SMAD2/3 and IGF-1/Akt/PI3K pathways.

Recent studies have proven that ncRNAs play an essential role in muscle atrophy during cancer cachexia through myostatin pathways, such as miR-23, miR-499, miR-206, miR-208a/b, and miR-27a [59,92,93].

It is found that miR-23a and miR-27a can attenuate SMAD signaling to inhibit the expression of SMAD2/3, myostatin RNA, and protein, slowing down the development of muscle atrophy [35,94]. It is worth noticing that miR-23a targets TRI63/MuRF and FBXO32/Atrogin-1 to suppress increased muscular dystrophy during cancer cachexia. Ji-Xia Kuang et al. have demonstrated that miR-185-5p [48] affects the myostatin-mediated signaling/SMAD3 pathway to reduce myotube atrophy, which increases the breakdown of proteins regulated by Atrogin-1 and MuRF-1.

Besides, it is reported that myostatin reduced the level of miR-486-5p, which can stimulate AKT signaling and reduce the PTEN and FoxO1a expression. In C2C12 cells, downregulated myostatin increased miR-486-5p levels and alleviated muscle loss [95].

### 3.6. SDF1/CXCR4 Pathway

The SDF1/CXCR4 pathway is increasingly being connected to muscle atrophy during cancer cachexia by mediating atrophy-associated protein degradation [96]. The cachectic muscle consistently displayed downregulation of three genes in this pathway: SDF1, ADCY7, and PAK1.

CXCR4 is SDF1’s receptor, and when activated, it can enhance protein content by reducing proteolysis. Furthermore, implications for CXCR4 in muscle differentiation have been observed in vitro, and a negative correlation exists between CXCR4 and atrogin-1/MuRF1 [96]. Relevant evidence has demonstrated that activating the CXCR4/CXCL12 pathway can preserve muscle mass, whereas the specific mechanism whereby the SDF1/CXCR4 pathway causes muscle atrophy needs to be researched [96].

**Figure 2:**
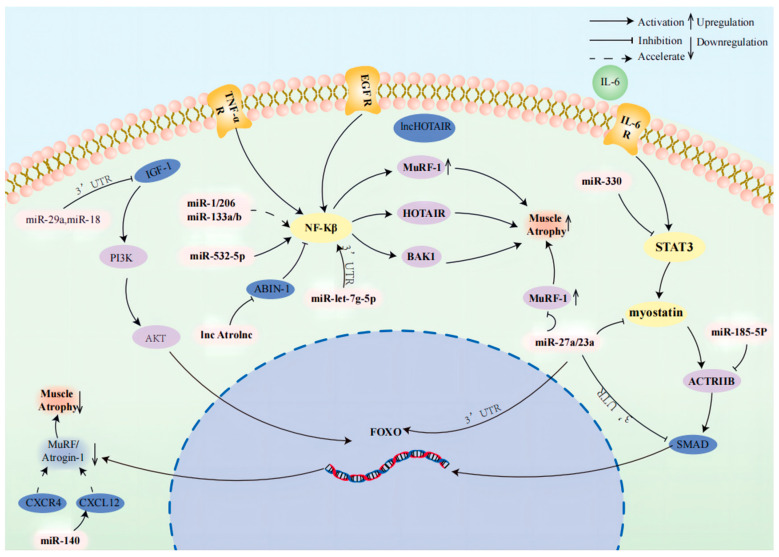
miR-18a targets IGF-1 3′ UTR to repress IGF-1 expression and, through PI3K-AKT-FOXO signaling, reduce muscle atrophy. miR-1, miR-206, miR-133a/b, lnc Atrolnc, miR-532-5p, and miR-let-7g-5p through the NF-Kβ pathway cause muscle atrophy. miR-330 causes muscle atrophy by affecting myostatin and STAT3 pathways. miR-27a and miR-23a directly affect MuRF-1 and restrain myostatin to cause muscle atrophy. The overexpression of miR-185-5p promotes muscle atrophy via the SMAD pathway.

An integrative meta-analysis study has revealed that miR-140 downregulated CXCL12 expression to be involved in muscular atrophy during cancer cachexia [9]. However, the mechanism of how miR-140 modulates CXCL12 to slow down atrophy progress is necessary to be explored in vivo and in vitro. Here is a compilation of how relevant non-coding RNAs regulate cachexia through muscle protein degradation (Figure 2, Table 2).

## 4. Mechanisms of Non-Coding RNA in Muscle Atrophy for Myoblasts

### 4.1. TGF-β/SMAD Pathway

Myoblast proliferation and differentiation are significant for regulating skeletal muscle atrophy, which is connected closely to the TGF-β and SMAD pathways [98]. The factor beta 1, which is involved in transforming growth, as a polypeptide signaling molecule, combines with type TGF-β receptors on the cell membrane and then activates type TGF-β receptors and transmits an extracellular signal into the cell [99]. Furthermore, endogenous TGF-β1dCas13b-FTO can target the demethylation of TGFβ1 mRNA, activating SMAD2 signaling [100]. As a result, the phosphorylation level of SMAD2 is upregulated, and myoblast proliferation is promoted. Besides, it is reported that m^6^A in TGF-β1 enhances its decay and suppresses its expression, bringing about the blockage of TGFβ1/SMAD2 signaling [100]. Except the SMAD2 relation to the TGF-β/SMAD pathway, TGF-β2/SMAD signaling associates with SMAD3, modulating target genes’ transcriptional activity as a complex combines with SMAD4 [101]. In addition, SMAD7 and SMURF1 can restrain the type I TGF-β receptor [102].

After long non-coding RNA SMUL is translated into protein [103], it disrupts the stability of SMURF2 mRNA via nonsense-mediated mRNA decay (NMD), which can inhibit SMURF2 expression and trigger TGF-β/SMAD signaling. Consequently, lncSMUL promotes myoblast proliferation and suppressed differentiation and induces skeletal muscle atrophy in vitro and in vivo. In addition, miR-22 [104] targets TGFBR1, the key receptor of the TGF-β/SMAD pathway, downregulates TGFBR1 expression, and decreases the Smad3 transmission of signals. Ultimately, C2C12 myoblast proliferation is suppressed, and myoblast differentiation into myotubes is promoted by the elevated miR-22.

In the skeletal muscle satellite cells (SMSCs), miR-200a-3p [101] targets TGF-β2 and decreases the phosphorylation of SMAD2 and SMAD3, promoting the differentiation and proliferation of SMSCs and meanwhile suppressing apoptosis of SMSCs. Therefore, miR-200a-3p improves the condition of skeletal muscle atrophy in chickens by regulating the TGF-β2/SMAD signaling pathway. In addition, miR-26a [105] was found to be widely expressed in mouse and human skeletal muscles. It can directly target SMAD1 and SMAD4 to regulate the TGF-b/bone morphogenetic proteins (BMP) signaling pathway, which connects closely with myogenesis and participates in the processes of cellular growth of skeletal muscle. When miR-26a is downregulated, the level of SMAD1/4 is reduced compared to previous levels, while muscle regeneration is retarded, and the level of differentiation decreases.

Roser Farre Garros et al. has discovered that miR-542-5p [106] expression can not only promote phosphorylation of SMAD2/3, thus enhancing muscle atrophy, but also inhibit the inhibitory components of TGF-β signaling from amplifying signal transduction of the TGF-β pathway. Moreover, it can improve the level of SMAD2/3 by inhibiting SMAD7 expression, which acts as an inhibitor of SMURF1, and compounds that inhibit type I TGF-β receptors. Even the phosphatases, limiting TGF-β signaling, were reduced by increasing the level of miR-542-5p.

Meanwhile, the expression of miR-422a [107] was found in the blood circulation of patients with COPD-induced muscle loss. The study revealed that miR-422a inhibited TGF-β signaling and repressed SMAD4 mainly through cell experiments and bioinformatics analysis, alleviating muscle loss in male patients with COPD. However, due to the lack of suitable samples, the exact level of SMAD4 protein in muscle biopsy samples from COPD patients was difficult to determine, and whether the inhibition of this protein is the main reason for miR-422a maintaining muscle mass cannot be confirmed. The mechanism of miR-422a opposing muscle atrophy by targeting SMAD4 needs more experiments in vivo and in vitro to be further proven.

### 4.2. Wnt/Notch Pathway

Satellite cells, quiescent muscle stem cells, are activated during muscle injury to differentiate into myoblasts, involved in the repair and regeneration of muscle tissue [108]. It has been shown that the differentiation of SCs is blocked in the skeletal muscle of cancer patients with muscle wasting, preventing the regeneration of myofibers [109]. Notch and Wnt signaling are commonly recognized to control cell fate decisions throughout embryonic development, essential in defining the tissue specialization of stem cells [110,111]. Moreover, Wnt promotes myoblast differentiation and myotube fusion, facilitating postnatal muscle regeneration [112,113,114]. In addition, Notch signaling in the skeletal muscle of sarcoma-bearing mice is overexpressed and contributes to the formation of muscular atrophy with increased TNF-α levels [115]. However, pro-inflammatory cytokine gene expression was not demonstrated to be higher in tumor samples from cachexia and non-cachexia groups in a different study [116]. Due to the complexities between C2C12, murine, and human models, more research should be carried out on the involvement of Wnt and Notch in cancer cachexia.

According to a recent study, the Wnt5a protein, shown to be a target of miR-487b in lung cancer cells, is regulated by a new long non-coding RNA called MAR1 (muscle anabolic regulator 1, MAR1), acting as the sponge of miR-487b [117] to promote muscular reconstruction.

The Gtl2-Dio3 locus, the most extensive known mammalian microRNA cluster, is immediately mediated by the transcription factor MEF2A. A portion of the Gtl2-Dio3miRNAs suppresses the WNT signaling inhibitors known as secreted frizzled-related proteins (sFRPs), which in turn stimulates myogenic differentiation [118]. In skeletal muscle knocked out of Mef2a, the level of sFRP2 elevated, while WNT expression downregulated. The study found that the reorganization of WNT3a and WNT5a could restore the differentiation in Mef2a-insufficient myoblasts. Moreover, the upregulated miR-433 and miR-410 (from the Gtl2-Dio3 locus) served a similar role by inhibiting sFRP2 [118].

Additionally, a temporal lag occurs between the production of Notch3 early in differentiation and myoblast stimulation by MyoD, as well as terminal differentiation into myotubes instructed by the myogenic transcription factor Mef2c. Mef2c triggers microRNAs miR-1 and miR-206, which straightly inhibit Notch3 and facilitate differentiation [119].

### 4.3. Other Regulators

#### 4.3.1. MyoD/MyoR/MEF2

MyoD affects cachexia muscle atrophy by influencing the growth, development, and metabolism of skeletal muscle [120] as one of the myogenic regulatory factors (MRFs) [121], which are crucial for initiating muscle cell differentiation and muscle growth, like myocyte enhancer factor 2 (MEF2) [122]. In terms of skeletal muscle growth and development, MyoD affects the differentiation of myogenic cells and the synthesis of related proteins. Once MyoD is deficient, myoblasts may transform into brown adipocytes [123]. MyoD also regulates skeletal muscle metabolism to maintain adequate energy for muscle contraction [124,125].

It is known that the development of skeletal muscle depends on transcriptional processes, which the bHLH (basic helix-loop-helix) family mediates [126]. MyoD is a bHLH transcription factor, which drives the activation of muscle specific gene expression by binding to bHLH protein. Conversely, MyoR suppresses myogenesis once combined with bHLH protein as a transcriptional repressor [127].

Some studies have confirmed that lncRNA muscle growth-promoting factor (lncMGPF) is observed to act as a molecular sponge of miR-135a-5p [122]. It restrains the level of miR-135a-5p, promoting MEF2C expression and increasing MyoD stability, enhancing myogenic differentiation and muscle growth. LncMGPF achieves these changes mainly through post-transcriptional regulation. As a result, the miR-135a-5p/MEF2C axis is an important pathway in the lncRNA-mediated network of myogenesis regulation. Researchers also found that pigs and humans have lncRNAs homologous to mouse lncMGPF, which positively regulate myogenesis.

It is shown that MyoD raised miR-378 [121] levels in C2C12 cell differentiation, and miR-378 contributes to the activation of MyoD by targeting its inhibitor, MyoR. However, in mice mules during regeneration after cardiotoxin injection, the significantly increased MyoD levels occurred earlier than the recovery of miR-378 levels, and MyoD levels decreased when miR-378 increased. Therefore, this suggests that MyoD is not the sole regulatory factor of miR-378 in skeletal muscle regeneration.

Zong-Kang Zhang et al. detected that overexpressing miR-762 in C2C12 cells led to downregulated expression of MyoD, and the formation of myotubes also maintained at a low level compared to normal conditions. They found an lncRNA mechanical unloading induced muscle atrophy-related (Lnc MUMA), which acts as a sponge for miR-762 and inhibits miR-762 in vitro to promote muscle differentiation, attenuating the progress of muscle atrophy [128].

#### 4.3.2. IGFII

Serving as an embryonic controller of myogenic processes, IGF-II controls the initiation of skeletal myogenesis and acts as an autocrine factor triggering myoblast differentiation in vitro [129]. It is reported that miR-125b is regulated by mTOR signaling and targets the IGFII-3′ UTR, negatively controlling muscle regeneration in vivo and myoblast differentiation in vitro [130].

#### 4.3.3. TLR7

Viral single-stranded RNA (ssRNA) sequences on B lymphocytes and dendritic cells may be identified and bound by members of the toll-like receptor (TLR) family, including human TLR8 and murine TLR7 [131], resulting in activating the expression of cells and cytokines, particularly miR-21 and miR-29a in exosomes derived from lung cancer cells. They bind to human macrophages’ toll-like receptor 8 (TLR8), which is homologous to TLR7 in mice, to provoke a proinflammatory response that promotes the growth of tumors [132].

Studies indicate that miR-21 from pancreatic and lung cancer cell lines triggers skeletal muscle apoptosis. Specifically, miR-21 released by MVs stimulates the murine myoblast TLR7 receptor and induces the cell death of muscular myoblasts via the effect of c-Jun N-terminal kinase (JNK) [133].

#### 4.3.4. PAX7

The previous study revealed that cancer cachexia is associated with a proliferation of SCs by observing an elevated production of the satellite cell marker Pax7 in cachexia models [134]. By reacting to NF-κB, the capacity of stem cells to differentiate in the muscle milieu was impeded via the constant expression of Pax7, which prevented them from merging with injured myofibers, leading to exacerbating muscle atrophy in cachexia [134].

MiR-431 directly targets Pax7 to drive satellite-cell variety during muscle growth and regeneration. Particularly, miR-431 may be a viable therapeutic option in muscular illnesses since it improves the symptoms of myodystrophy in mdx mice [135]. Besides, by limiting the ability of satellite cells to proliferate, miR-1 and miR-206 improve their differentiation, as Pax7 is a primary regulated target of miR-1/miR-206 [136]. Further studies are needed to identify the fundamental mechanism of miRs regulating cachexia muscular atrophy.

#### 4.3.5. S1PR3

According to a study [137], through stimulating SCs’ differentiation, upregulated miR-127 greatly improves myofibers’ reconstruction and lowers muscle degeneration in mdx animals. Furthermore, miR-127 specifically targets the S1PR3 (sphingosine-1-phosphate receptor 3) gene. In the mdx animal model, genetic excision of S1PR3 dramatically hindered the process of muscular atrophy, suggesting that S1PR3 may inhibit muscle differentiation [138]. Consequently, one prospective treatment approach for muscle atrophy could involve utilizing miR-127 to alter the S1PR3 or S1P expression.

#### 4.3.6. SRF

Serum response factor (SRF) is a transcription factor involved in muscle cell proliferation, differentiation, and migration [139]. A study found that the levels of SRF and miR-1 in the skeletal muscle of COPD patients were reduced compared with the healthy control group. When miR-1 expression decreased, SRF and its co-activators MRTFs were downregulated, and HDAC4 protein as another target of MiR-1 was increased, leading to COPD-related skeletal muscle atrophy and dysfunction [140].

**Figure 3:**
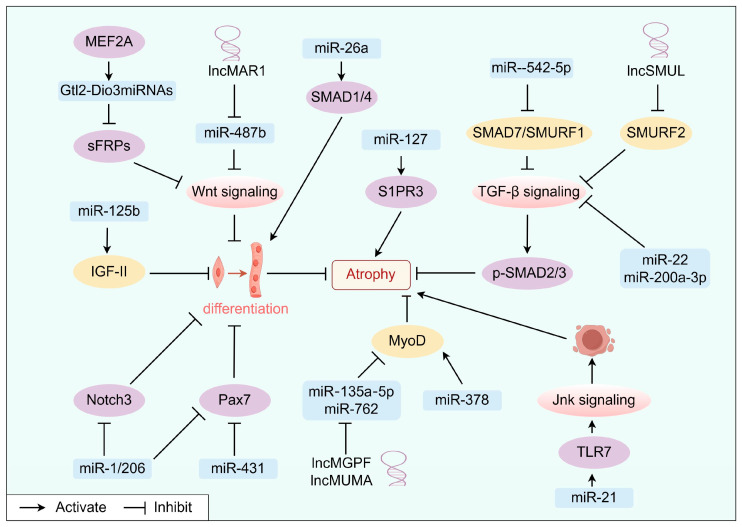
MiR-542-5p inhibits SMAD7/SMURF1, lncSMUL inhibits SMURF2, and miR-22/miR-200a-3p can inhibit TGF-β signaling to improve muscle atrophy. Gtl2-Dio3miRNAs suppress the WNT signaling via regulating sFRPs, and lncMAR1 acts as the sponge of miR-487b to promote muscular reconstruction. LncMGPF (the sponge of miR-135a-5p), lncMUMA (the sponge of miR-762), and miR-378 improve the MyoD. MiR-21 targets TLR7 to trigger muscle apoptosis. MiR-125b targets IGF-II, and miR-431 and miR-1/206 target Pax7 to control myoblast differentiation negatively. MiR-127 regulates S1PR3 to affect muscle differentiation.

Below is a collation of how specific ncRNAs modulate muscle cachexia by affecting myoblasts (Figure 3, Table 3).

## 5. Other Pathway

### ZIP4–CREB–miR-373–PHLPP2

ZIP4 is crucial in cancer-related cachexia of pancreatic cancer, which triggers the activation of CREB, a zinc-dependent transcription factor, stimulating p38 MAPK and FoxO expression by promoting the release of extracellular vesicles (EVs) [141].

It was found that overexpressing miR-373 in C2C12 myotubes upregulated muscle atrophy marker expression, and in cachexia with pancreatic cancer, ZIP4 activated miR-373 by increasing phosphorylated CREB [142]. ZIP4/miR-373 axis can target and combine with PHLPP2, causing the deactivation of AKT by regulating a phosphatase for AKT-Ser473. In ZIP4 knockout pancreatic cancer cells, the phosphorylation level of AKT consistently decreased [142]. Interestingly, Xiuhui Shi et al. noticed that a new circular RNA CircANAPC7, as a sponge for miR-373, can regulate the expression of PHLLP2 and phosphorylation of CREB, resulting in downregulated p-AKT, and prevent cancer cachectic muscle atrophy development. Furthermore, they found that CircANAPC7 can also downregulate TGF-β expression through dephosphorylation of STAT5 [142]. The study confirmed that the signaling axis involving ZIP4, miR-373, PHLLP2, and CircANAPC7 plays a remarkable role in pancreatic cancer and relevant cachexia muscle atrophy, suggesting that CircANAPC7 has the potential to be utilized in pancreatic cancer targeted therapy.

## 6. Non-Coding RNAs as a Potential Clinical Application for Cachexia Muscle Atrophy

A growing number of studies have reported the important role of ncRNAs in the early diagnosis of specific cachexia initiated by organ-specific diseases through liquid biopsy or solid biopsy and have suggested that ncRNAs will be potential targets for novel cachexia therapy [84,143].

### 6.1. ncRNAs as Diagnostic Markers of Cachexia

As a representative microRNA indicator of cachexia, miR-21 was upregulated in colon carcinoma, lung cancer, and pancreatic cancer patients suffering from muscular atrophy, promoting tumor growth and invasion as well as enhancing muscle loss [144,145]. It is worth mentioning that in serum from cachexia patients who have gastrointestinal cancer, lower expression of miR-122-5p related to more severe muscle atrophy, and miR-375 and miR-27b-3p were identified playing the same role as miR-122-5p in patients’ muscle tissue [80]. MiR-424-5p is an outstanding microRNA in several types of cachexia, which increases in COPD, breast cancer, endometrial cancer, and NSCLC patients to accelerate the progress of muscle atrophy [20,21,22], and miR-155 is also closely linked to cachexia induced by NSCLC or pancreatic cancer [84]. Meanwhile, the level of miR-345-5p increases significantly in skeletal muscle tissue from colorectal and pancreatic cancer patients [27], and raised miR-373 is also connected to pancreatic cancer-induced cachexia [142]. MiR-5682 level is positively related to the degree of LC-induced cachexia [79]. Interestingly, the rising plasma miR-499/miR-1 [74] and decreasing miR-422a [107] are related to muscle loss in COPD patients, while miR-122 and miR-16 levels do not change between patients and the healthy group [74].

It is reported that the level of lncRNA HOTAIR increases in cachexia induced by bladder cancer, and inhibition of lncRNA HOTAIR can decelerate cachectic progress [71]. Similarly, the elevated expression of lncRNA Atrolnc-1 in CKD or cancer mice indicated the development of cachexia [70]. However, more experiments focusing on human subjects are needed. Conversely, the low expression of lncRNA DRAIC has been linked to the poor condition of prostate cancer and glioblastoma [51]. Due to their stability, specificity, and sensitivity in liquid or solid biopsies, ncRNAs are highly potential diagnostic biomarkers of multiple types of cachectic muscle atrophy, which gastrointestinal cancer, pancreatic cancer, NSCLC, COPD, etc., induce. Certainly, the diagnostic evaluation of ncRNAs in many other types of cachexia needs to be explored further.

### 6.2. ncRNAs as Potential Drugs in Cachexia

Due to the ability to target specific molecules and regulate multiple signaling pathways, the potential of ncRNAs as therapeutic drugs for cachexia, especially induced by cancer, is gradually gaining attention. The most direct way to alleviate cancer cachexia is to prevent cancer progression; although there are no drugs based on ncRNAs in clinical cancer treatment currently, some relevant clinical trials have completed the first phase (e.g., NCT02369198) or are ongoing (e.g., NCT06307249, NCT02508090). The main approach for ncRNAs targeted treatment of cancer cachexia is that miRNA can directly bind to mRNA 3′ UTR, leading to mRNA degradation or preventing its translation process, affecting various signaling pathways. Conversely, lncRNA and circRNA can act as molecular sponges for miRNA, reducing mRNA degradation induced by miRNA. In accomplished clinical trial NCT02369198, TargomiR, an intravenous drug containing miR-16 mimetics, targeted EGFR in lung cancer cells to reverse the development of malignant pleural mesothelioma (MPM) and NSCLC [146]. At the same time, some trials were forced to be suspended due to severe immune reactions in patients [147]. We can find that during drug development, it is crucial to increase the precision of miR targeting molecules, reduce miR interference with its sponges, and determine the optimal therapeutic dose of miR and delivery methods [148]. In the treatment of cancer cachexia with miR drugs, both prospects and challenges coexist; therefore, in-depth research is needed continuously.

## 7. Conclusions and Perspectives

The growing research has recognized that non-coding RNAs are crucial regulators of muscle atrophy in cachexia, leading to the endeavor to elucidate the underlying mechanisms. As mentioned above, ncRNAs’ signaling pathways in multiple types of cachexia are currently being studied in depth, including pathways involving muscle protein synthesis, degradation, and various physiological stages of myoblasts. The intricacy of ncRNAs and complicated factors that cause target gene upregulation or downregulation are also gradually being revealed in vitro and in vivo, with the intention of identifying the specific triggers of this phenomenon of sustained skeletal muscle loss that is difficult to reverse. By clarifying the specific mechanisms by which ncRNAs affect muscle atrophy during cachexia, we might be able to find potential targets and identify relevant therapeutic strategies for this huge clinical challenge.

However, most related studies based on in vitro and animal models might not fully reflect the complex molecular interactions of cachexia in humans. Besides, the partial regulatory mechanisms of ncRNAs between cachexia and muscle atrophy initiated by aging, denervation, and poor nutrition are different; therefore, more research is needed to distinguish the functions of relevant signaling pathways in them. The understanding that ncRNAs impact specific tissues or organs and knowledge of their interaction during cachexia promotes the identification of more diagnostic and prognostic indicators of cachectic muscle atrophy, leading to early treatment interventions and higher quality of life for patients. Meanwhile, more studies are concentrating on targeted therapies in cachexia to regulate the expression of ncRNAs, especially miRNAs, by direct combination, regulation of miRNAs transcription, epigenetics, upstream genes, ceRNAs, intervention of upstream and downstream signals, and management of medications assembly methods and delivery systems. It is worth noting that cachexia is a systemic, comprehensive disease; ncRNAs drug development must consider integrating a series of aspects involving sustained safety, stability of curative effect, precise mechanisms, and pharmacology.

Future studies can further investigate how non-coding RNAs impact cachexia muscle atrophy by one or more specific signaling pathways and perform quantitative research to determine which type of ncRNA or combinations of several ncRNAs could be the optimal therapeutic targets or diagnostic markers, with the aim of developing safe and effective treatment for cachexia muscle atrophy.

## Figures and Tables

**Table 2:** Roles of non-coding RNAs in muscle protein degradation in cachectic muscle atrophy.

Pathway	ncRNAs	Type of Study	Time	Results	Biological Significance	References
PI3K -AKT -FOXO	miR-18a	In vitro: C2C12 myoblasts	/	Increased MuRF1, Atrophy-1, and CTSL expression	Downregulated the IGF-1 mRNA, and phosphorylation of Akt/FoxO3	[62]
	miR-21	In vitro: C57Bl/6 mice	3 d	FOXO3 translocated into the nucleus	Accelerated cachexia muscle atrophy	[63]
	miR-23a/27a	In vitro: Sham/CKD/diabetic mice	4 wk	Decreased the activation of AKT, FOXO1, and PTEN, reduced phosphorylation of SMAD3 and myostatin level	Reversed CKD-induced catabolic responses, regulated SMAD, PI3K/AKT signaling, increased dry weight and grip strength	[35]
NF-κB	miR-532-3p	In vitro: human skeletal myoblasts	/	Increased BAK1	Induced apoptosis and muscle atrophy	[73]
	lncRNA Atrolnc-1	In vivo: CKD mice; In vitro: C2C12 myotubes	3 wk	Increased MuRF-1, upregulated NF-κB signaling	Accelerated protein degradation in muscle cells	[70]
	lncRNA HOTAIR	In vivo: mice with MBT-2 tumor In vitro: bladder cancer cells, C2C12 myoblasts	30 d	Increased IL-6, TNF-α, and MuRF-1 activation	Reduced myotube diameter, accelerated muscle atrophy	[71]
	miR-1, miR-206, miR-133a/b	In vitro: Human skeletal muscle myoblasts	48 h	Decreased TNF-α signaling	Accelerated myotube formation	[85]
IL-6-JAK- STAT 3	miR-497-5p	In vitro: C2C12 mouse myoblasts	5 d	Increased IGF-1r and Insr expression	Accelerated atrophy of C2C12 myotubes	[48]
	miR-21	In vitro: C57Bl/6 mice, C2C12 myotubes	/	Overexpressed by IL-6 and TNF-α	Accelerated cell death of primary myoblasts	[63]
TNF-α	miR-155	In vitro: venous blood sample from NSCLC and pancreatic cancer patients	/	Targeted upstream genes of TNF-α (SOCS1 and Foxp3)	Accelerated cancer cachectic muscle atrophy	[84]
Myostatin	miR-486-5p	In vivo: SAMP8 mice; In vitro: C2C12 myoblasts	8 wk; 3 d	Myostatin inhibited miR-486-5p, decreased phosphorylation of AKT	Inhibited the transcription of MuRF1 and Atrogin-1 genes, FoxO1 level	[95]
	miR-183-5p	In vitro: C2C12 myoblasts	28 d	Increased Smad3, myostatin, Atrogin-1, and MuRF-1 expression	Enhanced myostatin, activated Smad3 signaling pathway, increased protein degradation	[97]
	miR-499/ miR-208a	In vitro: human muscle biopsies samples	/	Decreased myostatin and MEF2C	Inhibited myostatin to alleviate muscle atrophy	[59]

**Table 3:** Roles of non-coding RNAs in muscle atrophy for myoblasts.

Pathway/ Regulators	ncRNAs	Type of Study	Time	Results	Biological Significance	References
TGF-b/ SMAD	lncRNA SMUL	In vitro: chicken primary myoblast In vivo: chick gastrocnemius muscle	17 d	Decreased the SMURF2 mRNA and protein, SMURF2 downregulated TGF-β1 expression, and inhibited SMAD2/3 phosphorylation	Decreased myogenic differentiation and induced skeletal muscle atrophy Activated proteasomal degradation and autophagy	[103]
	miR-22	In vitro: Balb/c mice myoblasts	8 d	Inhibited TGFBR1, and miR-22 expression was down-regulated by TGF-β1	Inhibited myoblast proliferation and attenuated muscle atrophy	[104]
	MiR-200a-3p	In vitro/in vivo: breast muscle of broilers, layers at embryonic	11 d	Decreased p-SMAD2/3 expression, and exogenous TGF-β2 negatively regulates miR-200a-3p	Increased cell differentiation and suppressed apoptosis in skeletal muscle	[101]
	miR-26a	In vitro: C2C12 myoblasts	7 d	Downregulated the TGF-β/BMP signaling pathway, targets Smad1/4	Promoted muscle differentiation and regeneration	[105]
	miR-542	In vitro: LHCN-M2 cells In vivo: mice	/	Increased SMAD2/3 phosphorylation, suppresses SMAD7	Enhanced muscle atrophy	[106]
	miR-422a	In vitro: C2C12 myoblasts	/	Inhibited TGF-β signaling	Increased cell differentiation	[107]
Wnt	lncRNA MAR1	In vivo: C57BL/6J mice In vitro: C2C12 myoblasts	2 mnth	Increased Wnt5a, MAR1, MyoD, MyoG, Mef2c, and Myf5	Promoted skeletal muscle mass/strength	[117]
	Gtl2-Dio3 miRNAs locus	In vitro: C2C12 cells	/	Downregulated Mef2a and Wnt, increased sFRPs	Impaired myotube formation	[118]
Notch	miR-1, miR-206	In vitro: C2C12 myoblasts	48 h	Downregulated Notch3, upregulated Mef2c	Attenuated myoblast differentiation	[119]
	miR-378	In vitro: C2C12 myoblasts	48 h	Modulated MyoD activity by repressing MyoR	Promoted muscle differentiation	[121]
	lnc MGPF	In vitro/in vivo: mice/pig muscle cells	/	Inhibited miR-135a-5p, increased Mef2c and MyoD	Promoted myogenic differentiation	[122]
	Lnc MUMA	In vitro: C2C12 myoblasts	71 d	Elevated myoD level as a sponge for miR-762	Promoted muscle differentiation, muscle fiber cross-sectional area, and muscle strength	[128]
IGF-II	miR-125b	In vitro/in vivo: mice	2/3 d	Targeted the IGFII-3′ UTR	Inhibited myoblast differentiation	[130]
TLR7	miR-21	In vitro: TLR7−/− and TLR7+/+ mice myoblasts	48 h	Increased TLR7+/+ myoblasts death rate	Triggered apoptosis of muscle progenitor cells	[133]
PAX7	miR-431	In vivo: C57BL/6, TG mice; In vitro: C2C12 cells	1, 3, 5 and 7 d	Downregulated PAX7, increased SCs	Promoted muscle regeneration	[135]
	miR-1/ miR-206	In vitro: SCs, C2C12 cells	/	Decreased miR-1/miR-206, upregulated PAX7	Reinforced SCs from proliferation to differentiation	[136]
S1PR3	miR-127	In vivo: C57BL/6, TG mice; In vitro: C2C12 cells	2 wk	Downregulated S1PR3	Increased the size of myofibers and enhanced muscle regeneration	[137]
SRF	miR-1	In vitro: skeletal muscle with COPD patients	/	Downregulated SRF, MRTFs, and HDAC4 expression	Enhanced muscle atrophy and dysfunction	[140]

## Data Availability

Not applicable.

## Abbreviations

ncRNAs: on-coding RNAs; miRNAs: microRNAs; lncRNAs: long non-coding RNAs; circRNAs: circular RNAs; COPD: chronic obstructive pulmonary disease; IGF-1: insulin-like growth factor 1; IRS1: insulin receptor substrate-1; 4E-BP: 4E-binding proteins; S6K: S6 kinase; NSCLC: non-small cell lung cancer; PTEN: phosphatase and tensin homolog; MAFbx: muscle atrophy F-box; MuRF1: muscle RING finger 1; CKD: chronic kidney disease; DCM: diabetic cardiomyopathy; ceRNA: competing endogenous RNA; Cn: calcineurin; NFATc3: nuclear factor of activated T cells 3; DMD: Duchenne muscular dystrophy; AMPKs: AMP-activated protein kinases; TSC2: tuberous sclerosis complex 2; RHEB: Ras homolog enriched in brain; RAPTOR: regulatory associated protein of mTOR; GLUT1: glucose transporter 1; eIFs: eukaryotic initiation factors; SOX6: sex-determining region Y-box 6; UPS: ubiquitin proteasome system; ALS: autophagy-lysosome system; NIK: NF-κB-inducible kinase; EGFR: epidermal growth factor receptor; BAK1: BCL2 antagonist/killer 1; LC: laryngeal cancer; C/EBPδ: CCAAT/enhancer-binding protein δ; TWEAK: TNF-like weak inducer of apoptosis; TGF-β: transforming growth factor-β; ACTRIIB: activin receptor type IIB; NMD: nonsense-mediated mRNA decay; BMP: bone morphogenetic proteins; SCs: satellite cells; MAR1: muscle anabolic regulator 1; sFRPs: secreted frizzled-related proteins; MEF2: myocyte enhancer factor 2; bHLH: basic helix-loop-helix; MRFs: myogenic regulatory factors lncMGPF: lncRNA muscle growth-promoting factor; lnc MUMA: mechanical unloading induced muscle atrophy-related lncRNA; ssRNA: single-stranded RNA; TLR: toll-like receptor; S1PR3: sphingosine-1-phosphate receptor 3; S1P: sphingosine-1-phosphate; SRF: serum response factor; EVs: extracellular vesicles; MPM: malignant pleural mesothelioma

## References

[1] Ferrer M., Anthony T.G., Ayres J.S., Biffi G., Brown J.C., Caan B.J., Feliciano E.M.C., Coll A.P., Dunne R.F., Goncalves M.D. (2023). Cachexia: A systemic consequence of progressive, unresolved disease. Cell.

[2] Morena F., Cabrera A.R., Greene N.P. (2024). Exploring heterogeneity: A dive into preclinical models of cancer cachexia. Am. J. Physiol. Cell Physiol..

[3] Schmidt S.F., Rohm M., Herzig S., Berriel Diaz M. (2018). Cancer cachexia: More than skeletal muscle wasting. Trends Cancer.

[4] Setiawan T., Sari I.N., Wijaya Y.T., Julianto N.M., Muhammad J.A., Lee H., Chae J.H., Kwon H.Y. (2023). Cancer cachexia: Molecular mechanisms and treatment strategies. J. Hematol. Oncol..

[5] Argiles J.M., Busquets S., Stemmler B., Lopez-Soriano F.J. (2014). Cancer cachexia: Understanding the molecular basis. Nat. Rev. Cancer.

[6] Cabili M.N., Trapnell C., Goff L., Koziol M., Tazon-Vega B., Regev A., Rinn J.L. (2011). Integrative annotation of human large intergenic noncoding rnas reveals global properties and specific subclasses. Genes. Dev..

[7] von SHaehling N., Anker S.D., Haehling S. (2019). Recent developments in the field of cachexia, sarcopenia, and muscle wasting: Highlights from the 11th cachexia conference. J. Cachexia Sarcopenia Muscle.

[8] Ebner N., von Haehling S. (2018). Silver linings on the horizon: Highlights from the 10th cachexia conference. J. Cachexia Sarcopenia Muscle.

[9] Freire P.P., Fernandez G.J., Cury S.S., de Moraes D., Oliveira J.S., de Oliveira G., Dal-Pai-Silva M., dos Reis P.P., Carvalho R.F. (2019). The pathway to cancer cachexia: Microrna-regulated networks in muscle wasting based on integrative meta-analysis. Int. J. Mol. Sci..

[10] Liu Q., Deng J., Qiu Y., Gao J., Li J., Guan L., Lee H., Zhou Q., Xiao J. (2021). Non-coding rna basis of muscle atrophy. Mol. Ther. Nucleic Acids.

[11] Martone J., Mariani D., Desideri F., Ballarino M. (2019). Non-coding rnas shaping muscle. Front. Cell Dev. Biol..

[12] Nie M., Deng Z.L., Liu J., Wang D.Z. (2015). Noncoding rnas, emerging regulators of skeletal muscle development and diseases. Biomed Res. Int..

[13] Martin A., Gallot Y.S., Freyssenet D. (2023). Molecular mechanisms of cancer cachexia-related loss of skeletal muscle mass: Data analysis from preclinical and clinical studies. J. Cachexia Sarcopenia Muscle.

[14] Rommel C., Bodine S., Clarke B.A., Rossman R., Nunez L., Stitt T.N., Yancopoulos G.D., Glass D.J. (2001). Mediation of igf-1-induced skeletal myotube hypertrophy by pi(3)k/akt/mtor and pi(3)k/akt/gsk3 pathways. Nat. Cell Biol..

[15] Yoshida T., Delafontaine P. (2020). Mechanisms of igf-1-mediated regulation of skeletal muscle hypertrophy and atrophy. Cells.

[16] Tan Y., Shen L., Gan M., Fan Y., Cheng X., Zheng T., Niu L., Chen L., Jiang D., Li X. (2020). Downregulated mir-204 promotes skeletal muscle regeneration. Biomed Res. Int..

[17] Hu Y., Liu L., Chen Y., Zhang X., Zhou H., Hu S., Li X., Li M., Li J., Cheng S. (2023). Cancer-cell-secreted mir-204-5p induces leptin signalling pathway in white adipose tissue to promote cancer-associated cachexia. Nat. Commun..

[18] Li X., Qiu J., Liu H., Deng Y., Hu S., Hu J., Wang Y., Wang J. (2020). Microrna-33a negatively regulates myoblast proliferation by targeting igf1, follistatin and cyclin d1. Biosci. Rep..

[19] Connolly M., Paul R., Farre-Garros R., Natanek S.A., Bloch S., Lee J., Lorenzo J.P., Patel H., Cooper C., Sayer A.A. (2018). Mir-424-5p reduces ribosomal rna and protein synthesis in muscle wasting. J. Cachexia Sarcopenia Muscle.

[20] van de Worp W.R., Schols A.M., Dingemans A.C., Kamp C.M.O.D., Degens J.H., Kelders M.C., Coort S., Woodruff H.C., Kratassiouk G., Harel-Bellan A. (2020). Identification of micrornas in skeletal muscle associated with lung cancer cachexia. J. Cachexia Sarcopenia Muscle.

[21] Dastmalchi N., Hosseinpourfeizi M.A., Khojasteh S., Baradaran B., Safaralizadeh R. (2020). Tumor suppressive activity of mir-424-5p in breast cancer cells through targeting pd-l1 and modulating pten/pi3k/akt/mtor signaling pathway. Life Sci..

[22] Wang P., Liu T., Zhao Z., Wang Z., Liu S., Yang X. (2021). Sptbn2 regulated by mir-424-5p promotes endometrial cancer progression via cldn4/pi3k/akt axis. Cell Death Discov..

[23] Ni Q., Zhang H., Shi X., Li X. (2023). Exosomal lncrna hcg18 contributes to cholangiocarcinoma growth and metastasis through mediating mir-424-5p/sox9 axis through pi3k/akt pathway. Cancer Gene Ther..

[24] Mousa N.O., Sayed A.A., Fahmy N., Elzayat M.G., Bakry U., Abdellatif A., Zahra W.K., Osman A. (2021). Mirnome profiling in duchenne muscular dystrophy; Identification of asymptomatic and manifesting female carriers. Biosci. Rep..

[25] Cheng T.Y., Wu M.S., Hua K.T., Kuo M.L., Lin M.T. (2014). Cyr61/ctgf/nov family proteins in gastric carcinogenesis. World J. Gastroenterol..

[26] Zhang Y., Pan Q., Zhong H., Merajver S.D., Kleer C.G. (2005). Inhibition of ccn6 (wisp3) expression promotes neoplastic progression and enhances the effects of insulin-like growth factor-1 on breast epithelial cells. Breast Cancer Res..

[27] Narasimhan A., Ghosh S., Stretch C., Greiner R., Bathe O.F., Baracos V., Damaraju S. (2017). Small rnaome profiling from human skeletal muscle: Novel mirnas and their targets associated with cancer cachexia. J. Cachexia Sarcopenia Muscle.

[28] Song C., Yang Z., Dong D., Xu J., Wang J., Li H., Huang Y., Lan X., Lei C., Ma Y. (2019). Mir-483 inhibits bovine myoblast cell proliferation and differentiation via igf1/pi3k/akt signal pathway. J. Cell. Physiol..

[29] Li J., Chan M.C., Yu Y., Bei Y., Chen P., Zhou Q., Cheng L., Chen L., Ziegler O., Rowe G.C. (2017). Mir-29b contributes to multiple types of muscle atrophy. Nat. Commun..

[30] Dillmann W.H. (2019). Diabetic cardiomyopathy. Circ. Res..

[31] Yang X., Li X., Lin Q., Xu Q. (2019). Up-regulation of microrna-203 inhibits myocardial fibrosis and oxidative stress in mice with diabetic cardiomyopathy through the inhibition of pi3k/akt signaling pathway via pik3ca. Gene.

[32] Lee Y.R., Chen M., Pandolfi P.P. (2018). The functions and regulation of the pten tumour suppressor: New modes and prospects. Nat. Rev. Mol. Cell Biol..

[33] Wada S., Kato Y., Okutsu M., Miyaki S., Suzuki K., Yan Z., Schiaffino S., Asahara H., Ushida T., Akimoto T. (2011). Translational suppression of atrophic regulators by microrna-23a integrates resistance to skeletal muscle atrophy. J. Biol. Chem..

[34] Small E.M., O’rourke J.R., Moresi V., Sutherland L.B., McAnally J., Gerard R.D., Richardson J.A., Olson E.N. (2010). Regulation of pi3-kinase/akt signaling by muscle-enriched microrna-486. Proc. Natl. Acad. Sci. USA.

[35] Wang B., Zhang C., Zhang A., Cai H., Price S.R., Wang X.H. (2017). Microrna-23a and microrna-27a mimic exercise by ameliorating ckd-induced muscle atrophy. J. Am. Soc. Nephrol..

[36] Hudson M.B., Woodworth-Hobbs M.E., Zheng B., Rahnert J.A., Blount M.A., Gooch J.L., Searles C.D., Price S.R. (2014). Mir-23a is decreased during muscle atrophy by a mechanism that includes calcineurin signaling and exosome-mediated export. Am. J. Physiol. Cell Physiol..

[37] Lin Z., Murtaza I., Wang K., Jiao J., Gao J., Li P.F. (2009). Mir-23a functions downstream of nfatc3 to regulate cardiac hypertrophy. Proc. Natl. Acad. Sci. USA.

[38] Hudson M.B., Price S.R. (2013). Calcineurin: A poorly understood regulator of muscle mass. Int. J. Biochem. Cell Biol..

[39] Shen X., Tang J., Jiang R., Wang X., Yang Z., Huang Y., Lan X., Lei C., Chen H. (2021). Circrilpl1 promotes muscle proliferation and differentiation via binding mir-145 to activate igf1r/pi3k/akt pathway. Cell Death Dis..

[40] Zhao J., Zhao X., Shen X., Zhang Y., Zhang Y., Ye L., Li D., Zhu Q., Yin H. (2022). Circccdc91 regulates chicken skeletal muscle development by sponging mir-15 family via activating igf1-pi3k/akt signaling pathway. Poult. Sci..

[41] Li Z., Cai B., Abdalla B.A., Zhu X., Zheng M., Han P., Nie Q., Zhang X. (2019). Lncirs1 controls muscle atrophy via sponging mir-15 family to activate igf1-pi3k/akt pathway. J. Cachexia Sarcopenia Muscle.

[42] Elia L., Contu R., Quintavalle M., Varrone F., Chimenti C., Russo M.A., Cimino V., De Marinis L., Frustaci A., Catalucci D. (2009). Reciprocal regulation of microrna-1 and insulin-like growth factor-1 signal transduction cascade in cardiac and skeletal muscle in physiological and pathological conditions. Circulation.

[43] Huang M.B., Xu H., Xie S.J., Zhou H., Qu L.H. (2011). Insulin-like growth factor-1 receptor is regulated by microrna-133 during skeletal myogenesis. PLoS ONE.

[44] Li G., Li Q.-S., Li W.-B., Wei J., Chang W.-K., Chen Z., Qiao H.-Y., Jia Y.-W., Tian J.-H., Liang B.-S. (2016). Mirna targeted signaling pathway in the early stage of denervated fast and slow muscle atrophy. Neural Regen. Res..

[45] de Paula T.G., Zanella B.T.T., Fantinatti B.E.d.A., de Moraes L.N., Duran B.O.d.S., de Oliveira C.B., Salomão R.A.S., da Silva R.N., Padovani C.R., dos Santos V.B. (2017). Food restriction increase the expression of mtorc1 complex genes in the skeletal muscle of juvenile pacu (*Piaractus mesopotamicus*). PLoS ONE.

[46] Alexander M.S., Casar J.C., Motohashi N., Myers J.A., Eisenberg I., Gonzalez R.T., Estrella E.A., Kang P.B., Kawahara G., Kunkel L.M. (2011). Regulation of dmd pathology by an ankyrin-encoded mirna. Skelet. Muscle.

[47] Hitachi K., Nakatani M., Tsuchida K. (2014). Myostatin signaling regulates akt activity via the regulation of mir-486 expression. Int. J. Biochem. Cell Biol..

[48] Freire P.P., Cury S.S., Lopes L.O., Fernandez G.J., Liu J., de Moraes L.N., de Oliveira G., Oliveira J.S., de Moraes D., Cabral-Marques O. (2021). Decreased mir-497-5p suppresses il-6 induced atrophy in muscle cells. Cells.

[49] Herzig S., Shaw R.J. (2018). Ampk: Guardian of metabolism and mitochondrial homeostasis. Nat. Rev. Mol. Cell Biol..

[50] Egan D., Kim J., Shaw R.J., Guan K.L. (2011). The autophagy initiating kinase ulk1 is regulated via opposing phosphorylation by ampk and mtor. Autophagy.

[51] Saha S., Zhang Y., Wilson B., Abounader R., Dutta A. (2021). The tumor-suppressive long noncoding rna draic inhibits protein translation and induces autophagy by activating ampk. J. Cell Sci..

[52] Jackson R.J., Hellen C.U., Pestova T.V. (2010). The mechanism of eukaryotic translation initiation and principles of its regulation. Nat. Rev. Mol. Cell Biol..

[53] Brito Q.J., Diaz-Lopez I., Ramakrishnan V. (2024). The molecular basis of translation initiation and its regulation in eukaryotes. Nat. Rev. Mol. Cell Biol..

[54] Liang R., Shen X., Wang F., Wang X., DesJarlais A., Syed A., Saba R., Tan Z., Yu F., Ji X. (2021). H19x-encoded mir-322(424)/mir-503 regulates muscle mass by targeting translation initiation factors. J. Cachexia Sarcopenia Muscle.

[55] Pardo P.S., Hajira A., Boriek A.M., Mohamed J.S. (2017). Microrna-434-3p regulates age-related apoptosis through eif5a1 in the skeletal muscle. Aging.

[56] Soares R.J., Cagnin S., Chemello F., Silvestrin M., Musaro A., De Pitta C., Lanfranchi G., Sandri M. (2014). Involvement of micrornas in the regulation of muscle wasting during catabolic conditions. J. Biol. Chem..

[57] Khachigian L.M. (2018). The yin and yang of yy1 in tumor growth and suppression. Int. J. Cancer.

[58] Li J., Yang T., Tang H., Sha Z., Chen R., Chen L., Yu Y., Rowe G.C., Das S., Xiao J. (2021). Inhibition of lncrna maat controls multiple types of muscle atrophy by cis- and trans-regulatory actions. Mol. Ther..

[59] Drummond M.J., Glynn E.L., Fry C.S., Dhanani S., Volpi E., Rasmussen B.B. (2009). Essential amino acids increase microrna-499, -208b, and -23a and downregulate myostatin and myocyte enhancer factor 2c mrna expression in human skeletal muscle. J. Nutr..

[60] You J.-S., Dooley M.S., Kim C.-R., Kim E.-J., Xu W., Goodman C.A., Hornberger T.A. (2018). A dgkzeta-foxo-ubiquitin proteolytic axis controls fiber size during skeletal muscle remodeling. Sci. Signal.

[61] Stitt T.N., Drujan D., Clarke B.A., Panaro F., Timofeyva Y., Kline W.O., Gonzalez M., Yancopoulos G.D., Glass D.J. (2004). The igf-1/pi3k/akt pathway prevents expression of muscle atrophy-induced ubiquitin ligases by inhibiting foxo transcription factors. Mol. Cell.

[62] Liu C., Wang M., Chen M., Zhang K., Gu L., Li Q., Yu Z., Li N., Meng Q. (2017). Mir-18a induces myotubes atrophy by down-regulating igfi. Int. J. Biochem. Cell Biol..

[63] Borja-Gonzalez M., Casas-Martinez J.C., McDonagh B., Goljanek-Whysall K. (2020). Inflamma-mir-21 negatively regulates myogenesis during ageing. Antioxidants.

[64] Chen B., Gao T., Yuan W., Zhao W., Wang T.H., Wu J. (2019). Prognostic value of survival of micrornas signatures in non-small cell lung cancer. J. Cancer.

[65] Che J., Xu C., Wu Y., Jia P., Han Q., Ma Y., Wang X., Zheng Y. (2021). Mir-1290 promotes myoblast differentiation and protects against myotube atrophy via akt/p70/foxo3 pathway regulation. Skelet. Muscle.

[66] Sakuma K., Aoi W., Yamaguchi A. (2017). Molecular mechanism of sarcopenia and cachexia: Recent research advances. Pflügers Arch. Eur. J. Physiol..

[67] Li Y.P., Schwartz R.J., Waddell I.D., Holloway B.R., Reid M.B. (1998). Skeletal muscle myocytes undergo protein loss and reactive oxygen-mediated nf-kappab activation in response to tumor necrosis factor alpha. Faseb J..

[68] Di Marco S., Mazroui R., Dallaire P., Chittur S., Tenenbaum S.A., Radzioch D., Marette A., Gallouzi I.-E. (2005). Nf-kappa b-mediated myod decay during muscle wasting requires nitric oxide synthase mrna stabilization, hur protein, and nitric oxide release. Mol. Cell. Biol..

[69] Fry C.S., Nayeem S.Z., Dillon E.L., Sarkar P.S., Tumurbaatar B., Urban R.J., Wright T.J., Sheffield-Moore M., Tilton R.G., Choudhary S. (2016). Glucocorticoids increase skeletal muscle nf-kappab inducing kinase (nik): Links to muscle atrophy. Physiol. Rep..

[70] Sun L., Si M., Liu X., Choi J.M., Wang Y., Thomas S.S., Peng H., Hu Z., Sun L., Si M. (2018). Long-noncoding rna atrolnc-1 promotes muscle wasting in mice with chronic kidney disease. J. Cachexia Sarcopenia Muscle.

[71] Hu C.-Y., Su B.-H., Lee Y.-C., Wang C.-T., Yang M.-L., Shen W.-T., Fu J.-T., Chen S.-Y., Huang W.-Y., Ou C.-H. (2022). Interruption of the long non-coding rna hotair signaling axis ameliorates chemotherapy-induced cachexia in bladder cancer. J. Biomed. Sci..

[72] Tsai C.H., Huang P.J., Lee I.T., Chen C.M., Wu M.H. (2022). Endothelin-1-mediated mir-let-7g-5p triggers interlukin-6 and tnf-alpha to cause myopathy and chronic adipose inflammation in elderly patients with diabetes mellitus. Aging.

[73] Chen F.X., Shen Y., Liu Y., Wang H.F., Liang C.Y., Luo M. (2020). Inflammation-dependent downregulation of mir-532-3p mediates apoptotic signaling in human sarcopenia through targeting bak1. Int. J. Biol. Sci..

[74] Donaldson A., Natanek S.A., Lewis A., Man W.D.-C., Hopkinson N.S., Polkey M.I., Kemp P.R. (2013). Increased skeletal muscle-specific microrna in the blood of patients with copd. Thorax.

[75] Bonetto A., Aydogdu T., Jin X., Zhang Z., Zhan R., Puzis L., Koniaris L.G., Zimmers T.A. (2012). Jak/stat3 pathway inhibition blocks skeletal muscle wasting downstream of il-6 and in experimental cancer cachexia. Am. J. Physiol. Endocrinol. Metab..

[76] Mubaid S., Ma J.F., Omer A., Ashour K., Lian X.J., Sanchez B.J., Robinson S., Cammas A., Dormoy-Raclet V., Di Marco S. (2019). Hur counteracts mir-330 to promote stat3 translation during inflammation-induced muscle wasting. Proc. Natl. Acad. Sci. USA.

[77] Mazurek M., Brzozowska A., Maziarz M., Malecka-Massalska T., Powrozek T. (2024). The relationship between mir-5682 and nutritional status of radiotherapy-treated male laryngeal cancer patients. Genes.

[78] Liu Z., Song Y.N., Chen K.Y., Gao W.L., Chen H.J., Liang G.Y. (2022). Bioinformatics prediction of potential mechanisms and biomarkers underlying dilated cardiomyopathy. World J. Cardiol..

[79] Wu Q., Liu Z., Li B., Liu Y.E., Wang P. (2024). Immunoregulation in cancer-associated cachexia. J. Adv. Res..

[80] Krauss T., Heisz S., Honecker J., Prokopchuk O., Martignoni M., Janssen K., Claussnitzer M., Hauner H., Seeliger C. (2023). Specific mirnas are associated with human cancer cachexia in an organ-specific manner. J. Cachexia Sarcopenia Muscle.

[81] Carbó N., López-Soriano J., Costelli P., Busquets S., Alvarez B., Baccino F.M., Quinn L.S., López-Soriano F.J., Argilés J.M. (2000). Interleukin-15 antagonizes muscle protein waste in tumour-bearing rats. Br. J. Cancer.

[82] Pistilli E.E., Siu P.M., Alway S.E. (2007). Interleukin-15 responses to aging and unloading-induced skeletal muscle atrophy. Am. J. Physiol. Cell Physiol..

[83] Patel H.J., Patel B.M. (2017). Tnf-alpha and cancer cachexia: Molecular insights and clinical implications. Life Sci..

[84] Yehia R., Schaalan M., Abdallah D.M., Saad A.S., Sarhan N., Saleh S. (2021). Impact of tnf-alpha gene polymorphisms on pancreatic and non-small cell lung cancer-induced cachexia in adult egyptian patients: A focus on pathogenic trajectories. Front. Oncol..

[85] Georgantas R.W., Streicher K., Greenberg S.A., Greenlees L.M., Zhu W., Brohawn P.Z., Higgs B.W., Czapiga M., Morehouse C.A., Amato A. (2014). Inhibition of myogenic micrornas 1, 133, and 206 by inflammatory cytokines links inflammation and muscle degeneration in adult inflammatory myopathies. Arthritis Rheumatol..

[86] Iannone F., Montesanto A., Cione E., Crocco P., Caroleo M.C., Dato S., Rose G., Passarino G. (2020). Expression patterns of muscle-specific mir-133b and mir-206 correlate with nutritional status and sarcopenia. Nutrients.

[87] Powrozek T., Pigon-Zajac D., Mazurek M., Ochieng O.M., Rahnama-Hezavah M., Malecka-Massalska T. (2022). Tnf-alpha induced myotube atrophy in c2c12 cell line uncovers putative inflammatory-related lncrnas mediating muscle wasting. Int. J. Mol. Sci..

[88] Sharma M., McFarlane C., Kambadur R., Kukreti H., Bonala S., Srinivasan S. (2015). Myostatin: Expanding horizons. Iubmb Life.

[89] Trendelenburg A.U., Meyer A., Rohner D., Boyle J., Hatakeyama S., Glass D.J. (2009). Myostatin reduces akt/torc1/p70s6k signaling, inhibiting myoblast differentiation and myotube size. Am. J. Physiol. Cell Physiol..

[90] Zhu X., Topouzis S., Liang L.F., Stotish R.L. (2004). Myostatin signaling through smad2, smad3 and smad4 is regulated by the inhibitory smad7 by a negative feedback mechanism. Cytokine.

[91] Lokireddy S., Mouly V., Butler-Browne G., Gluckman P.D., Sharma M., Kambadur R., McFarlane C. (2011). Myostatin promotes the wasting of human myoblast cultures through promoting ubiquitin-proteasome pathway-mediated loss of sarcomeric proteins. Am. J. Physiol. Cell Physiol..

[92] Callis T.E., Pandya K., Seok H.Y., Tang R.-H., Tatsuguchi M., Huang Z.-P., Chen J.-F., Deng Z., Gunn B., Shumate J. (2009). Microrna-208a is a regulator of cardiac hypertrophy and conduction in mice. J. Clin. Investig..

[93] Han L., Li P., He Q., Yang C., Jiang M., Wang Y., Cao Y., Han X., Liu X., Wu W. (2023). Revisiting skeletal muscle dysfunction and exercise in chronic obstructive pulmonary disease: Emerging significance of myokines. Aging Dis..

[94] Zhang A., Li M., Wang B., Klein J.D., Price S.R., Wang X.H. (2018). Mirna-23a/27a attenuates muscle atrophy and renal fibrosis through muscle-kidney crosstalk. J. Cachexia Sarcopenia Muscle.

[95] Chang Y.C., Liu H.W., Chan Y.C., Hu S.H., Liu M.Y., Chang S.J. (2020). The green tea polyphenol epigallocatechin-3-gallate attenuates age-associated muscle loss via regulation of mir-486-5p and myostatin. Arch. Biochem. Biophys..

[96] Martinelli G.B., Olivari D., Cecconi A.D.R., Talamini L., Ottoboni L., Lecker S.H., Stretch C., E. Baracos V., Bathe O.F., Resovi A. (2016). Activation of the sdf1/cxcr4 pathway retards muscle atrophy during cancer cachexia. Oncogene.

[97] Kuang J., Shen Q., Zhang R., Fang Q., Deng X., Fan M., Cheng C., Zhang X., Liu X. (2022). Carnosol attenuated atrophy of c2c12 myotubes induced by tumour-derived exosomal mir-183-5p through inhibiting smad3 pathway activation and keeping mitochondrial respiration. Basic Clin. Pharmacol. Toxicol..

[98] Li X., McFarland D.C., Velleman S.G. (2008). Effect of smad3-mediated transforming growth factor-beta1 signaling on satellite cell proliferation and differentiation in chickens. Poult. Sci..

[99] Derynck R., Zhang Y.E. (2003). Smad-dependent and smad-independent pathways in tgf-beta family signalling. Nature.

[100] Deng K., Liu Z., Li X., Zhang Z., Fan Y., Huang Q., Zhang Y., Wang F. (2023). Targeted demethylation of the tgfβ1 mrna promotes myoblast proliferation via activating the smad2 signaling pathway. Cells.

[101] Yin H., He H., Shen X., Tang S., Zhao J., Cao X., Han S., Cui C., Chen Y., Wei Y. (2020). Microrna profiling reveals an abundant mir-200a-3p promotes skeletal muscle satellite cell development by targeting tgf-β2 and regulating the tgf-β2/smad signaling pathway. Int. J. Mol. Sci..

[102] Suzuki C., Murakami G., Fukuchi M., Shimanuki T., Shikauchi Y., Imamura T., Miyazono K. (2002). Smurf1 regulates the inhibitory activity of smad7 by targeting smad7 to the plasma membrane. J. Biol. Chem..

[103] Cai B., Li Z., Ma M., Zhang J., Kong S., Abdalla B.A., Xu H., Jebessa E., Zhang X., Lawal R.A. (2021). Long noncoding rna smul suppresses smurf2 production-mediated muscle atrophy via nonsense-mediated mrna decay. Mol. Ther. Nucleic Acids.

[104] Wang H., Zhang Q., Wang B., Wu W., Wei J., Li P., Huang R. (2018). Mir-22 regulates c2c12 myoblast proliferation and differentiation by targeting tgfbr1. Eur. J. Cell Biol..

[105] Dey B.K., Gagan J., Yan Z., Dutta A. (2012). Mir-26a is required for skeletal muscle differentiation and regeneration in mice. Genes Dev..

[106] Garros R.F., Paul R., Connolly M., Lewis A., Garfield B.E., Natanek S.A., Bloch S., Mouly V., Griffiths M.J., Polkey M.I. (2017). Microrna-542 promotes mitochondrial dysfunction and smad activity and is elevated in intensive care unit-acquired weakness. Am. J. Respir. Crit. Care Med..

[107] Paul R., Lee J., Donaldson A.V., Connolly M., Sharif M., Natanek S.A., Rosendahl U., Polkey M.I., Griffiths M., Kemp P.R. (2018). Mir-422a suppresses smad4 protein expression and promotes resistance to muscle loss. J. Cachexia Sarcopenia Muscle.

[108] Dumont N.A., Bentzinger C.F., Sincennes M.C., Rudnicki M.A. (2015). Satellite cells and skeletal muscle regeneration. Compr. Physiol..

[109] Brzeszczyńska J., Johns N., Schilb A., Degen S., Degen M., Langen R., Schols A., Glass D.J., Roubenoff R., Greig C.A. (2016). Loss of oxidative defense and potential blockade of satellite cell maturation in the skeletal muscle of patients with cancer but not in the healthy elderly. Aging.

[110] Artavanis-Tsakonas S., Rand M.D., Lake R.J. (1999). Notch signaling: Cell fate control and signal integration in development. Science.

[111] Tajbakhsh S., Borello U., Vivarelli E., Kelly R., Papkoff J., Duprez D., Buckingham M., Cossu G. (1998). Differential activation of myf5 and myod by different wnts in explants of mouse paraxial mesoderm and the later activation of myogenesis in the absence of myf5. Development.

[112] Polesskaya A., Seale P., Rudnicki M.A. (2003). Wnt signaling induces the myogenic specification of resident cd45^+^ adult stem cells during muscle regeneration. Cell.

[113] Mermelstein C.S., Portilho D.M., Mendes F.A., Costa M.L., Abreu J.G. (2007). Wnt/beta-catenin pathway activation and myogenic differentiation are induced by cholesterol depletion. Differentiation.

[114] Rochat A., Fernandez A., Vandromme M., Molès J.-P., Bouschet T., Carnac G., Lamb N.J.C. (2004). Insulin and wnt1 pathways cooperate to induce reserve cell activation in differentiation and myotube hypertrophy. Mol. Biol. Cell.

[115] Mu X., Agarwal R., March D., Rothenberg A., Voigt C., Tebbets J., Huard J., Weiss K. (2016). Notch signaling mediates skeletal muscle atrophy in cancer cachexia caused by osteosarcoma. Sarcoma.

[116] Lu F., Osei-Hwedieh D., Mandell J.B., Morales-Restrepo A., Hankins M.L., Crasto J.A., Ma R., Dinh V.V., Watters R.J., Weiss K.R. (2019). Comparison of cachectic and non-cachectic sarcoma patients reveals an important role of notch signaling in metastasis and myogenesis. Am. J. Cancer Res..

[117] Zhang Z., Li J., Guan D., Liang C., Zhuo Z., Liu J., Lu A., Zhang G., Zhang B. (2018). A newly identified lncrna mar1 acts as a mir-487b sponge to promote skeletal muscle differentiation and regeneration. J. Cachexia Sarcopenia Muscle.

[118] Snyder C.M., Rice A.L., Estrella N.L., Held A., Kandarian S.C., Naya F.J. (2013). Mef2a regulates the gtl2-dio3 microrna mega-cluster to modulate wnt signaling in skeletal muscle regeneration. Development.

[119] Gagan J., Dey B.K., Layer R., Yan Z., Dutta A. (2012). Notch3 and mef2c proteins are mutually antagonistic via mkp1 protein and mir-1/206 micrornas in differentiating myoblasts. J. Biol. Chem..

[120] Hu X., Sun M., Chen Q., Zhao Y., Liang N., Wang S., Yin P., Yang Y., Lam S.M., Zhang Q. (2023). Skeletal muscle-secreted dlpc orchestrates systemic energy homeostasis by enhancing adipose browning. Nat. Commun..

[121] Gagan J., Dey B.K., Layer R., Yan Z., Dutta A. (2011). Microrna-378 targets the myogenic repressor myor during myoblast differentiation. J. Biol. Chem..

[122] Lv W., Jin J., Xu Z., Luo H., Guo Y., Wang X., Wang S., Zhang J., Zuo H., Bai W. (2020). Lncmgpf is a novel positive regulator of muscle growth and regeneration. J. Cachexia Sarcopenia Muscle.

[123] Wang C., Liu W., Nie Y., Qaher M., Horton H.E., Yue F., Asakura A., Kuang S. (2017). Loss of myod promotes fate transdifferentiation of myoblasts into brown adipocytes. Ebiomedicine.

[124] Shintaku J., Peterson J.M., Talbert E.E., Gu J.-M., Ladner K.J., Williams D.R., Mousavi K., Wang R., Sartorelli V., Guttridge D.C. (2016). Myod regulates skeletal muscle oxidative metabolism cooperatively with alternative nf-kappab. Cell Rep..

[125] Hodge B.A., Zhang X., Gutierrez-Monreal M.A., Cao Y., Hammers D.W., Yao Z., Wolff C.A., Du P., Kemler D., Judge A.R. (2019). Myod1 functions as a clock amplifier as well as a critical co-factor for downstream circadian gene expression in muscle. Elife.

[126] Yu L., Mikloucich J., Sangster N., Perez A., McCormick P.J. (2003). Myor is expressed in nonmyogenic cells and can inhibit their differentiation. Exp. Cell Res..

[127] Lu J., Webb R., Richardson J.A., Olson E.N. (1999). Myor: A muscle-restricted basic helix-loop-helix transcription factor that antagonizes the actions of myod. Proc. Natl. Acad. Sci. USA.

[128] Zhang Z.-K., Li J., Guan D., Liang C., Zhuo Z., Liu J., Lu A., Zhang G., Zhang B.-T. (2018). Long noncoding rna lncmuma reverses established skeletal muscle atrophy following mechanical unloading. Mol. Ther..

[129] Florini J.R., Ewton D.Z., Coolican S.A. (1996). Growth hormone and the insulin-like growth factor system in myogenesis. Endocr. Rev..

[130] Ge Y., Sun Y., Chen J. (2011). Igf-ii is regulated by microrna-125b in skeletal myogenesis. J. Cell Biol..

[131] Heil F., Hemmi H., Hochrein H., Ampenberger F., Kirschning C., Akira S., Lipford G., Wagner H., Bauer S. (2004). Species-specific recognition of single-stranded rna via toll-like receptor 7 and 8. Science.

[132] Fabbri M., Paone A., Calore F., Galli R., Gaudio E., Santhanam R., Lovat F., Fadda P., Mao C., Nuovo G.J. (2012). Micrornas bind to toll-like receptors to induce prometastatic inflammatory response. Proc. Natl. Acad. Sci. USA.

[133] He W.A., Calore F., Londhe P., Canella A., Guttridge D.C., Croce C.M. (2014). Microvesicles containing mirnas promote muscle cell death in cancer cachexia via tlr7. Proc. Natl. Acad. Sci. USA.

[134] He W.A., Berardi E., Cardillo V.M., Acharyya S., Aulino P., Thomas-Ahner J., Wang J., Bloomston M., Muscarella P., Nau P. (2013). Nf-kappab-mediated pax7 dysregulation in the muscle microenvironment promotes cancer cachexia. J. Clin. Investig..

[135] Wu R., Li H., Zhai L., Zou X., Meng J., Zhong R., Li C., Wang H., Zhang Y., Zhu D. (2015). Microrna-431 accelerates muscle regeneration and ameliorates muscular dystrophy by targeting pax7 in mice. Nat. Commun..

[136] Chen J.-F., Tao Y., Li J., Deng Z., Yan Z., Xiao X., Wang D.-Z. (2010). Microrna-1 and microrna-206 regulate skeletal muscle satellite cell proliferation and differentiation by repressing pax7. J. Cell Biol..

[137] Zhai L., Wu R., Han W., Zhang Y., Zhu D. (2017). Mir-127 enhances myogenic cell differentiation by targeting s1pr3. Cell Death Dis..

[138] Fortier M., Figeac N., White R.B., Knopp P., Zammit P.S. (2013). Sphingosine-1-phosphate receptor 3 influences cell cycle progression in muscle satellite cells. Dev. Biol..

[139] Braun T., Gautel M. (2011). Transcriptional mechanisms regulating skeletal muscle differentiation, growth and homeostasis. Nat. Rev. Mol. Cell Biol..

[140] Lewis A., Riddoch-Contreras J., Natanek S.A., Donaldson A., Man W.D.-C., Moxham J., Hopkinson N.S., I. Polkey M., Kemp P.R. (2012). Downregulation of the serum response factor/mir-1 axis in the quadriceps of patients with copd. Thorax.

[141] Yang J., Zhang Z., Zhang Y., Ni X., Zhang G., Cui X., Liu M., Xu C., Zhang Q., Zhu H. (2019). Zip4 promotes muscle wasting and cachexia in mice with orthotopic pancreatic tumors by stimulating rab27b-regulated release of extracellular vesicles from cancer cells. Gastroenterology.

[142] Shi X., Yang J., Liu M., Zhang Y., Zhou Z., Luo W., Fung K.-M., Xu C., Bronze M.S., Houchen C.W. (2022). Circular rna anapc7 inhibits tumor growth and muscle wasting via phlpp2-akt-tgf-β signaling axis in pancreatic cancer. Gastroenterology.

[143] Belli R., Ferraro E., Molfino A., Carletti R., Tambaro F., Costelli P., Muscaritoli M. (2021). Liquid biopsy for cancer cachexia: Focus on muscle-derived micrornas. Int. J. Mol. Sci..

[144] Okugawa Y., Yao L., Toiyama Y., Yamamoto A., Shigemori T., Yin C., Omura Y., Ide S., Kitajima T., Shimura T. (2018). Prognostic impact of sarcopenia and its correlation with circulating mir-21 in colorectal cancer patients. Oncol. Rep..

[145] Calore F., Londhe P., Fadda P., Nigita G., Casadei L., Marceca G.P., Fassan M., Lovat F., Gasparini P., Rizzotto L. (2018). The tlr7/8/9 antagonist imo-8503 inhibits cancer-induced cachexia. Cancer Res..

[146] van Zandwijk N., Pavlakis N., Kao S.C., Linton A., Boyer M.J., Clarke S., Huynh Y., Chrzanowska A., Fulham M.J., Bailey D.L. (2017). Safety and activity of microrna-loaded minicells in patients with recurrent malignant pleural mesothelioma: A first-in-man, phase 1, open-label, dose-escalation study. Lancet Oncol..

[147] Hanna J., Hossain G.S., Kocerha J. (2019). The potential for microrna therapeutics and clinical research. Front. Genet..

[148] Sannicandro A.J., McDonagh B., Goljanek-Whysall K. (2020). Micrornas as potential therapeutic targets for muscle wasting during cancer cachexia. Curr. Opin. Clin. Nutr. Metab. Care.

